# Ethanol and caffeine age-dependently alter brain and retinal neurochemical levels without affecting morphology of juvenile and adult zebrafish *(Danio rerio)*

**DOI:** 10.1371/journal.pone.0286596

**Published:** 2023-07-05

**Authors:** Carly L. Clayman, Christina Hwang, Victoria P. Connaughton

**Affiliations:** Department of Biology and Center for Neuroscience and Behavior, American University, Washington, DC, United States of America; University Hospital Heidelberg: UniversitatsKlinikum Heidelberg, GERMANY

## Abstract

Adolescent alcohol exposure in humans is predictive of adult development of alcoholism. In rodents, caffeine pre-exposure enhances adult responsiveness to ethanol via a pathway targeted by both compounds. Embryonic exposure to either compound adversely affects development, and both compounds can alter zebrafish behaviors. Here, we evaluate whether co-exposure to caffeine and/or alcohol in adolescence exerts neurochemical changes in retina and brain. Zebrafish (*Danio rerio*) were given daily 20 min treatments to ethanol (1.5% v/v), caffeine (25–100 mg/L), or caffeine + ethanol for 1 week during mid-late adolescence (53–92 days post fertilization (dpf)) or early adulthood (93–142 dpf). Immediately after exposure, anatomical measurements were taken, including weight, heart rate, pigment density, length, girth, gill width, inner and outer eye distance. Brain and retinal tissue were subsequently collected either (1) immediately, (2) after a short interval (2-4d) following exposure, or (3) after a longer interval that included an acute 1.5% ethanol challenge. Chronic ethanol and/or caffeine exposure did not alter anatomical parameters. However, retinal and brain levels of tyrosine hydroxylase were elevated in fish sacrificed after the long interval following exposure. Protein levels of glutamic acid decarboxylase were also increased, with the highest levels observed in 70–79 dpf fish exposed to caffeine. The influence of ethanol and caffeine exposure on neurochemistry demonstrates specificity of their effects during postembryonic development. Using the zebrafish model to assess neurochemistry relevant to reward and anxiety may inform understanding of the mechanisms that reinforce co-addiction to alcohol and stimulants.

## Introduction

Alcohol and caffeine are some of the most commonly consumed drugs in the world [1], with significant numbers of adolescents aged 12–20 reporting intake of either compound [2,3]. Both chemicals are often consumed together with the idea that ethanol, as a stress-reducer, may offset the anxiogenic effects of caffeine as a form of self-medication [4]. Conversely, stimulant substances may moderate the impact of alcohol on neural circuit development to reciprocally enhance the rewarding aspects of both substances. For example, as an antagonist of the adenosine receptor, caffeine acts a neuromodulator, potentiating the effects of ethanol [5]. Alcohol, in contrast, inhibits adenosine re-uptake, contributing to increased extracellular adenosine levels and adenosine receptor activation.

Ethanol exposure in zebrafish affects locomotion, aggression, anxiety, social behavior [6,7], and the production of brain neurotransmitters [8]. Embryonic or larval exposure delays development of motor skills and reflexes by directly inhibiting motor neuron and muscle fiber formation [9]. Chronic ethanol exposure alters baseline behaviors in zebrafish, as well as behavioral responses to ethanol, suggesting neuroplastic changes. These underlying changes have implications for ethanol-induced CPP [10], alcohol withdrawal [11–14], the response to the novelty of ethanol, and/or changes in neurotransmitter systems [15–18]. Caffeine is similarly teratogenic to zebrafish, causing developmental abnormalities in the cardiovascular, neuromuscular, and nervous systems [19–21]. Caffeine increases adenosine receptor expression, has downstream effects on adenosine signal transduction, and alters brain-derived neurotrophic factor expression [22].

Ethanol’s sedative properties are attributed to its effects on the GABAergic system [23,24]. Acute embryonic alcohol exposure dose-dependently decreases dopamine (DA), GABA, and glutamate levels in strain AB zebrafish [15]. In contrast, SF strain fish show a dose-dependent increase in GABA levels with acute exposure [15]. Pre-exposure to caffeine in larval zebrafish and adolescent rodents influences subsequent adult response to ethanol through the action of DARPP-32, a protein kinase and phosphatase regulator targeted by both alcohol and caffeine [22,25,26]. Ethanol and caffeine increase dopaminergic signaling via adenosine and DA receptors in brain [27]. Caffeine also contributes to enhanced turnover of monoaminergic neurotransmitters and alters DA neurotransmission [28]. Enhanced striatal DA signaling is implicated in the synergistic effects of caffeine and ethanol on locomotor sensitization [29].

The purpose of this study was to identify changes in dopaminergic and GABAergic circuits in zebrafish repeatedly exposed to ethanol and/or caffeine. We assessed these neurochemical changes by examining changes in tyrosine hydroxylase (TH) and glutamic acid decarboxylase (GAD), the rate-limiting enzymes in dopamine and GABA synthesis, respectively. Changes in protein levels in brain and retinal tissue were assessed immediately after 7 days of exposure to ethanol and/or caffeine or control water, after a short withdrawal interval, or after a longer withdrawal interval. Given that adolescent alcohol exposure is predictive of adult development of alcoholism, fish were exposed during the juvenile, young adult, and adult stages to assess age-dependent and long-term effects. Our results reveal drug-dependent changes in TH and GAD protein levels in both brain and retina. These changes depended on age of exposure as well as duration of post-exposure interval. In contrast, anatomical changes, assessed immediately after exposure and prior to sacrificing fish for neurochemical assessments, were not altered by ethanol and/or caffeine exposure.

## Materials and methods

### Animals

Adult, wild-type zebrafish (*Danio rerio*) were maintained in the lab on a 14 hr light/10 hr dark photoperiod at 28–29 °C in an Aquatic Habitats (AHAB) rack system. Fish were obtained from a local pet store. Fish were monitored and fed daily when environmental parameters were measured. This study was carried out in strict accordance with the recommendations in the *Guide for the Care and Use of Laboratory Animals*. All procedures were approved by the Institutional Animal Care and Use Committee at American University (protocols #1304 and #1610). All efforts were made to minimize pain and suffering.

To obtain eggs/larvae, fish were separated by sex in their home tanks for at least two days prior to being placed into a breeding chamber containing 1–5 males and 1–5 females. Fish remained in the breeding chamber overnight and eggs were collected next day. Within 1–2 hr post fertilization (hpf), eggs were cleaned, staged, and placed in petri dishes of egg water (0.12 g instant ocean + 200 μL 0.1% methylene blue dissolved in 2 L of Deer Park (system) water). Petri dishes were maintained at the same temperature and photoperiod as stock tanks and water was changed daily to remove debris.

At 48 hpf, embryos hatched, and larvae were fed a pinch of finely ground AP-100 brine mix. Fish were transferred into the AHAB when they were between 1 and 2 weeks of age or when they had grown large enough to be safely placed in the recirculating system in mesh nursery enclosures. Fish were fed AP-100 twice daily once placed in the AHAB and supplemented with *Artemia* starting at 2 weeks of age.

### Drug exposure

Juvenile (52–79 dpf), young adult (80–99 dpf), or adult (100–169 dpf) fish of mixed sex were removed from AHAB tanks and immersed individually in a total volume of 10 mL (juveniles) or 50 mL (young adults and adults) of their respective daily 20 min treatments for 7 consecutive days, as in our previous study [35]. Fish were gently netted from their holding chamber containing AHAB system water and the net was inverted to place fish individually in their treatment container. Immediately following the 20 min treatment, the treatment container was inverted over a net to transfer fish into a new (wash) container prior to returning the fish to their holding container of fresh system water. Each fish was assigned to one of the following treatments using block randomization with each block being the treatment start date: (1) 1.5% (v/v; diluted from 100%/200 proof) ethanol, (2) 25 mg/L caffeine, (3) 25 mg/L caffeine + 1.5% ethanol, (4) 50 mg/L caffeine, (5) 50 mg/L caffeine + 1.5% ethanol, (6) 75 mg/L caffeine, (7) 75 mg/L caffeine + 1.5% ethanol, (8) 100 mg/L caffeine, (9) 100 mg/L caffeine + 1.5% ethanol, or (10) control water. All chemicals were purchased from Sigma.

Treatment exposures of more than one individual occurred simultaneously with fish placed in either 1 well of a 6-well plate (juvenile fish) or in plastic beakers (adult fish). Different solution volumes were used to account for differences in the size of the experimental containers and so all fish would have similar capacities for mobility. Once in experimental treatments, fish were assessed daily to control for direct effects of exposure. During exposures, experimental containers were housed in a water bath to maintain the same temperature and pH as the stock/holding tanks. No differences in pH were noted with the addition of either caffeine or ethanol to the treatment containers.

After the 7 day (d) exposure period, treated fish were either (1) euthanized in a 0.02% tricaine solution for anatomical measurement and tissue collection (immediate sacrifice after exposure), (2) transferred to control water for a short (2–4 d) withdrawal period before tissue collection, or (3) transferred to control water for a longer (28–60 d) withdrawal period that included an acute (1.5%) ethanol challenge prior to tissue collection. The experimenters were blinded to the animal’s group during subsequent weighing, anatomical measurements, neurochemical measurements, Image-J pixel density measurements, and statistical analysis.

### Anatomical assessment

Each fish was weighed (g) on a microbalance (Sartorius) and heart rate was measured by direct observation on an Olympus SX-61 stereomicroscope. There were 3 independent replicates per treatment condition (each ethanol/caffeine dose) for a total of 18 subjects. Heart rate was measured for 30 sec and then multiplied by 2 to obtain beats per minute. Fish were imaged using MetaMorph software (Olympus) and micrographs were taken of the dorsal, ventral, and lateral sides for subsequent anatomical measurements of total length (dorsal, ventral, lateral views), gill width (ventral), girth (ventral), and inner and outer eye distance. For all anatomical measurements, there were 7–9 independent replicates per treatment condition (each ethanol/caffeine dose) for a total of 51 subjects. Overall body pigmentation was also quantified. Measurements were taken from micrographs using NIH ImageJ.

Length measurements (dorsal, ventral, and lateral) were taken from the most cranial to the most caudal edge of each fish. Gill width was measured from the dorsal side, across the outermost edges of the gill arches. Girth was measured from the ventral side, across the outermost edges of the belly. Inner and outer eye distances were measured from the ventral side, across the innermost and outermost edges of the eyes, respectively. Pigmentation was measured using densitometry by first converting micrographs to 8-bit gray-scale. Then, a standard area measurement was designated for each group of fish (from which images were taken on the same day) and the average pigment density for the controls from each group of fish was determined. Treatment-induced changes in pigmentation were determined by normalizing pigment density of a given fish to the average density of the corresponding control group.

### Neurochemistry

Brain tissue and both retinas were collected from each fish. The brain was placed in 25 μL of RIPA buffer, two retinas were placed in 16 μL of RIPA, and then frozen at -80 °C for Western Blots. After homogenization and two centrifugation steps, the protein concentration of the supernatant was quantified using a Bradford assay as performed in other studies measuring zebrafish brain protein content [30]. Then, 20 μg of protein lysate in RIPA buffer was loaded into each lane of 2–15% gradient gels that were separated in the SDS-PAGE apparatus, as performed in previous studies [31,32].

TH (tyrosine hydroxylase) levels in brain and retinal tissue were quantified to determine differences in dopamine levels, as TH activity coincides with dopamine levels [33]. GAD65/67 (glutamic acid decarboxylase) was used as a measure of neuronal GABA levels, and GAPDH was the loading control. For all neurochemical measurements, there were 4–6 independent replicates per treatment condition (each ethanol/caffeine dose) for a total of 35 subjects. Antibody providers and dilutions were TH primary antibody: Millipore MAB318, 1:2000, with secondary anti-mouse antibody 1:1000; GAD65/67 primary antibody: Millipore AB1511, 1:1000, with secondary anti-rabbit antibody 1:1000; GAPDH primary antibody: Millipore ab8245, 1:500, with secondary anti-mouse antibody 1:1000. Secondary antibodies were purchased from Jackson ImmunoResearch.

Image J was utilized for pixel density quantification of bands, with densitometry of TH and GAD65/67 bands normalized to GAPDH prior to statistical analysis.

### Statistical analysis

#### Anatomical assessment

R statistical computing software was used to run a three-way ANOVA to identify differences across treatments. Outliers with values outside of 1.5 times the interquartile range within groups were excluded from analysis. As groups without at least 4 observations were excluded from morphology analysis, our analysis was confined to the 0, 25, and 100 mg/L caffeine doses and the 0% or 1.5% ethanol doses. When deviation from normality was present, as assessed by the Shapiro-Wilk test, or when homogeneity of variances was not present, as assessed by Levene’s test, a non-parametric Aligned Rank Transform (ART) ANOVA was applied to assess interaction and main effects. This non-parametric approach is used to assess three-way variable interactions when assumptions for an ANOVA are not met. The independent variables were ethanol dose (0% or 1.5%), caffeine dose (0 mg/L; 25 mg/L; or 100 mg/L), and post-exposure measurement age (60–79, 80–99, 100–119, 120–139, or 140–169 dpf). If significant main effects or interactions were identified, post hoc tests with Holm adjustment for multiple comparisons were performed.

#### Neurochemistry

R statistical computing software was used to run a three-way ANOVA to identify differences across treatments. Samples with GAPDH levels more than 2.5 times the standard error above or below the mean calculated per each blot were excluded from the analysis. Consequently, outliers with values outside of 1.5 times the interquartile range within groups were excluded. Neurochemical measurements were normalized against loading controls, and results were transformed using a log(10) transformation or 1/(x+1) transformation to address skewed data distributions and restore normality. In measures with persistent deviation from normality after transformations were implemented, an Aligned Rank Transform (ART) non-parametric ANOVA was applied to assess interaction and main effects. Our analysis was confined to the 0, 25, and 100 mg/L caffeine doses and the 0% or 1.5% ethanol doses. Exposure age and dissection (post-exposure) age were separated into bins for pairwise comparisons in post hoc tests. Ages were juvenile stages: 40–49, 50–59, and 60–69 dpf; young adult stages: 70–79, 80–89, and 90–99 dpf; adult stages: 100–109, 110–119, 120–129, 130–139, 140–149, and 150–159 dpf. Three-way ANOVA or ART-ANOVA was used to determine the impact of ethanol and/or caffeine exposure, and either age of exposure or age/time of dissection on neurochemical results. If significant main effects or interactions were identified, post hoc tests with Holm adjustment for multiple comparisons were performed. To account for the influence of the GAPDH loading control on statistical analysis, the densitometry values for GAPDH for each variable of interest (ethanol dose, caffeine dose, pre-exposure age, and time of sacrifice) on the same blot were compared using a one-way ANOVA. This analysis showed that GAPDH levels did not vary across each of these measures for brain and retina except for retinal GAPDH which varied along with time of sacrifice (S2 Fig).

## Results

### Anatomical assessment

**Fig 1:**
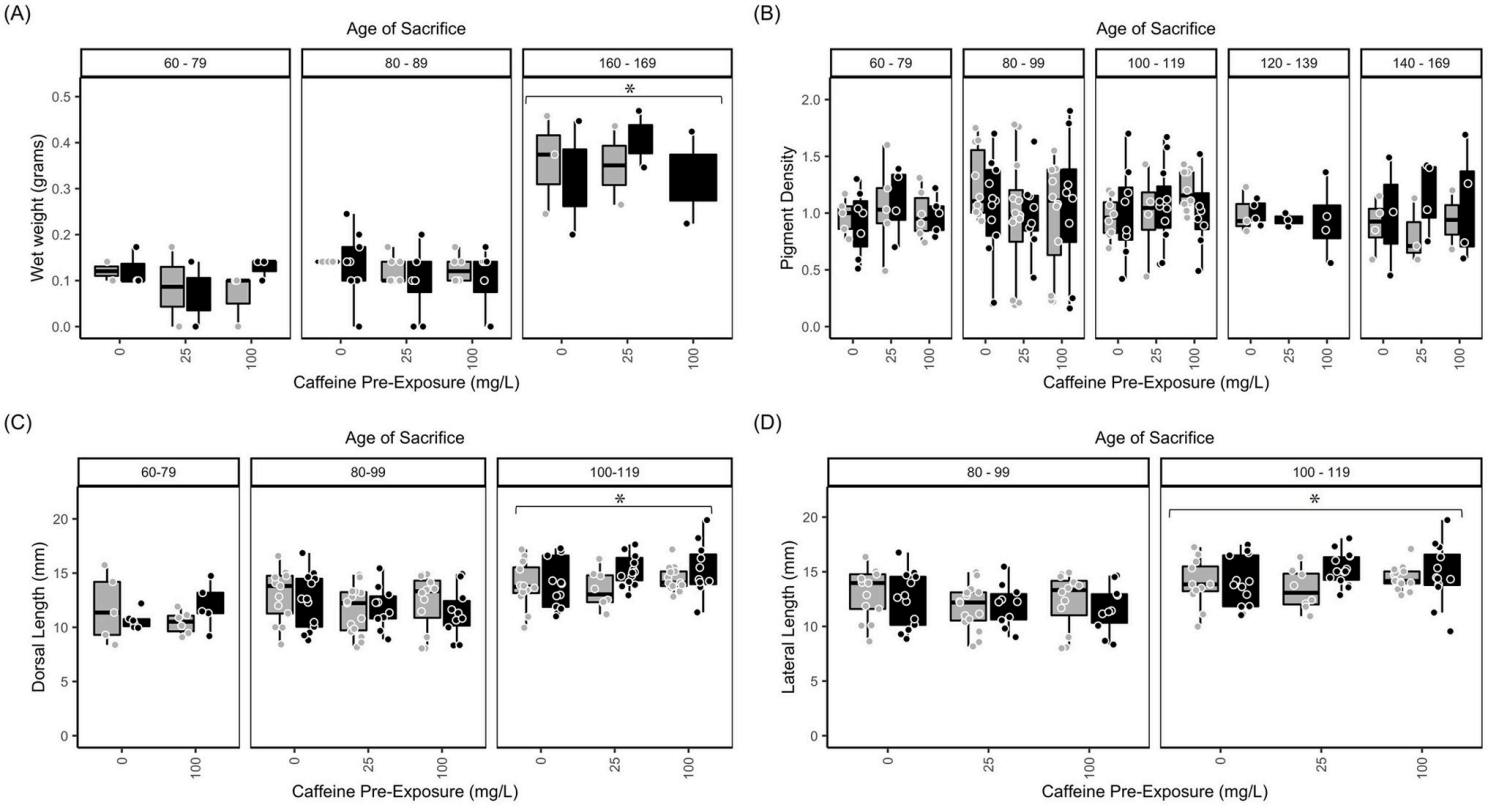
Acute 7-day exposure to caffeine and/or ethanol does not alter length or weight. (A) Wet weight (g), (B) pigment density, (C) dorsal length (mm), and (D) lateral length (mm) measurements of zebrafish co-exposed to caffeine and ethanol. Measurements were taken immediately after the 7-d exposure period. Measurements were pooled to assess the effect of age of sacrifice across these experimental conditions. Exposure ages (dpf) are indicated at the top of the bar graphs. Black bars = 1.5% ethanol; gray bars = control (0% ethanol). Caffeine exposures (0, 25, or 100 mg/L) are given along the x-axis. Data from individual fish were assessed in age bins to facilitate comparisons: 60–79 dpf (juveniles); 80–99 dpf (young adult); 100–119; 120–139; 140–169 dpf (adult). Wet weight was collected from 60–79 dpf, 80–89 dpf, and 160–169 dpf fish only, to identify the largest differences. Neither caffeine nor ethanol exposure affected measurements. However, age-dependent differences were noted (asterisks). The boxplots show the minimum, first quartile, median, third quartile, and maximum values for each measure after outliers were removed. For weight (A), there were 3 independent replicates per treatment condition (each ethanol/caffeine dose) for a total of 18 subjects. For all other parameters (B, C, D) there were 7–9 independent replicates per treatment condition for a total of 51 subjects. ANOVA tables and multiple comparison results for this data can be found in S1 Table, panels (A1, A2) weight; (B) pigment density; (C1, C2) Dorsal Length; (D1-D2) Sagittal (Lateral) Length.

Weight of fish measured at 160–169 dpf was significantly larger than the weight of all other groups (Fig 1A, p<0.0001). Body pigmentation was not significantly different across measurement ages or ethanol / caffeine pre-exposure doses (Fig 1B).

Adults measured at 100–119 dpf had a significantly larger dorsal length (Fig 1C) compared to all other groups (p<0.0001). Lateral length (Fig 1D) also differed significantly based on age of measurement. Fish measured at 100–119 dpf were significantly longer than fish measured at 80–99 dpf (p<0.0001).

**Fig 2:**
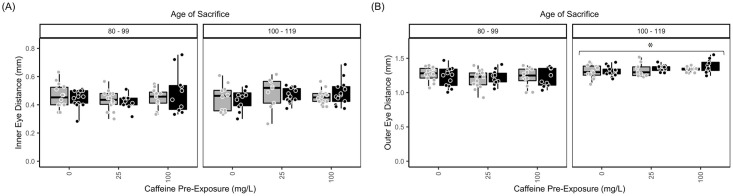
Age, but not ethanol and/or caffeine treatment, significantly affects eye diameter. As in Fig 1, measurements of (A) inner and (B) outer eye diameter (in mm) were taken immediately after the 7-d exposure period to ethanol and/or caffeine. Measurements were pooled to assess the effect of age of sacrifice across these experimental conditions. Black bars = 1.5% ethanol; gray bars = control (0% ethanol). Caffeine exposures (0, 25, or 100 mg/L) are given along the x-axis. Data from individual fish were assessed in age bins to facilitate comparisons: 80–99 dpf (young adult); 100–119 dpf (adult). Despite age-dependent differences (asterisks), ethanol and/or caffeine exposure did not significantly impact measurements. The boxplots show the minimum, first quartile, median, third quartile, and maximum values for each measure after outliers were removed. Age when sacrificed (dpf) is indicated at the top of the bar graphs. For both measurements, there were 7–9 independent replicates per treatment condition (each ethanol/caffeine dose) for a total of 51 subjects. ANOVA tables and multiple comparison results for this data can be found in S1 Table, panels (E) Inner Eye Distance; (F1, F2) Outer Eye Distance.

Examining the eye revealed that inner eye distance (Fig 2A) was not significantly changed due to measurement age. Outer eye distance (Fig 2B) was significantly larger when measured at 100–119 dpf compared to 80–99 dpf (p≤0.0001) No significant differences were observed for heart rate, gill width, or girth (data now shown).

The above data revealed that exposure condition/drug did not affect any of the anatomical endpoints. Rather, all significant differences were due to the age of the fish at the time of measurement. This suggests that 7 consecutive days of exposure to ethanol and/or caffeine during the juvenile or adult stage does not cause overt differences in anatomy.

### Neurochemistry

Multifactorial ANOVAs for parametric outcomes and ART ANOVAs for nonparametric outcomes were used to assess the effects of ethanol and/or caffeine exposure, age of exposure, and post-exposure (withdrawal) duration on brain (n = 144) and retinal (n = 243) TH levels. Values greater than 2.5 times the standard error above and below the mean per blot were excluded from analysis along with outliers and subjects from under-represented groups for both ANOVAs and plots. ANOVA tables are included in S1 Table. Western Blots are presented in S1 and S2 Figs.

#### Tyrosine hydroxylase—Brain

**Fig 3:**
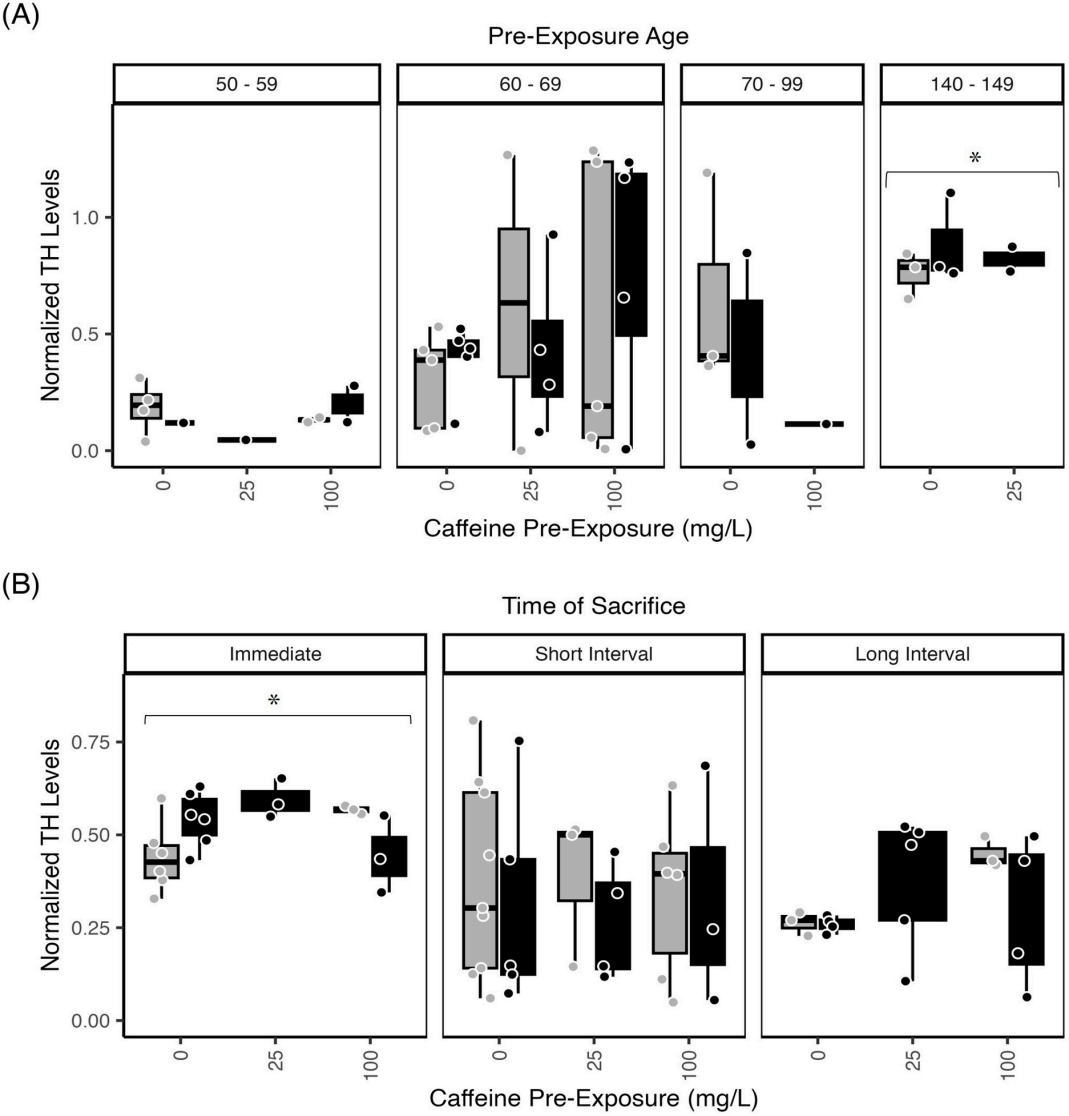
Zebrafish Brain TH levels increase with age and ethanol withdrawal. Normalized TH levels in brain homogenates from zebrafish treated with 1.5% ethanol (black bars) or water (control, gray bars) plotted across caffeine dose (0 mg/L; 25 mg/L, 100 mg/L). (A) TH levels in brain tissue assessed immediately after exposure. Fish exposed at 140–149 dpf had increased TH levels relative to other exposure ages (p<0.0.006; asterisk). (B) TH levels were also compared across time of sacrifice—immediately after exposure, after the short post-exposure interval, or after the long post-exposure interval. Tissue collected immediately had significantly greater TH levels compared to tissue collected after the short interval or long interval (p<0.0037; asterisk). The boxplots show the minimum, first quartile, median, third quartile, and maximum values for each measure after outliers were removed. Exposure ages (dpf) are indicated at the top of the bar graphs. For brain TH levels, there were 4–6 independent replicates per treatment condition (each ethanol/caffeine dose) for a total of 35 subjects. ANOVA tables and multiple comparison results for this data can be found in S1 Table, panels (G1, G2) Brain TH levels across pre-exposure age; (H1, H2) Brain TH levels across time of sacrifice.

Brain TH levels showed a significant effects of exposure age (Fig 3A), with subjects exposed at 140–149 dpf showing significantly greater brain TH, compared to subjects exposed from 50–59 dpf, 60–69 dpf, and 70–99 dpf (p<0.0.006). While this could be due to older fish having larger brains and, therefore, more brain protein, we do not think this is the case as we loaded the same amount of protein across lanes in the Westerns, all TH levels were normalized to GAPDH, and samples that had more than 2.5 times the standard error above and below the mean were excluded per each blot (S1 Fig).

We also note relevant trends in the data. Specifically, the differences between ethanol-exposed and ethanol-naïve subjects were typically exaggerated at higher caffeine doses. For example, brain TH levels in older adults (140–149 dpf) showed a trend of an increase following exposure to 25 and 100 mg/L caffeine compared to controls (Fig 3A). The trend of caffeine influencing the effect of ethanol exposure was also demonstrated in fish exposed at 60–69 dpf, with relatively greater levels of TH in the 100 mg/L caffeine group (Fig 3A).

Overall, there was a significant effect of time of sacrifice (p<0.021), with greater levels of brain TH in subjects sacrificed immediately following exposure compared to those subjects sacrificed after a long interval after exposure (p<0.0037) (Fig 3B).

#### Tyrosine hydroxylase—Retina

**Fig 4:**
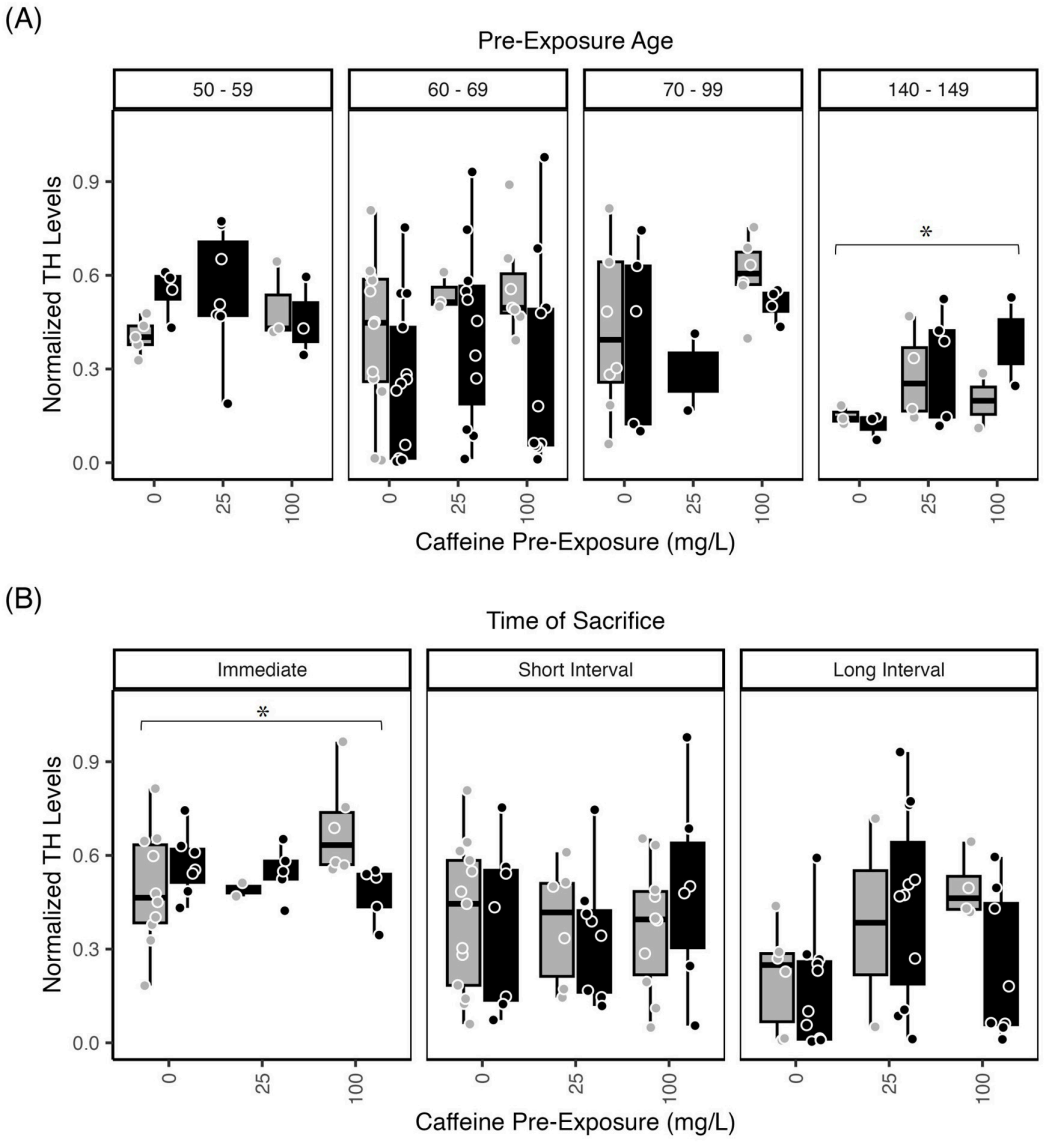
Zebrafish Retina TH levels are affected by age and ethanol withdrawal. Normalized retinal TH levels from zebrafish exposed to 1.5% ethanol (black bars) or water (control, grey bars) with or without caffeine (0 mg/L; 25 mg/L, 100 mg/L). (A) TH levels assessed immediately after exposure. Fish exposed at 50–59 dpf and 70–99 dpf had significantly higher retinal TH levels relative to 140–149 dpf (p<0.008; asterisk). (B) TH levels of retinal homogenates revealed significantly greater TH levels after the immediate post-exposure interval, compared to fish sacrificed after the short or long post-exposure interval (p<0.004; asterisk). The boxplots show the minimum, first quartile, median, third quartile, and maximum values for each measure after outliers were removed. Exposure ages (dpf) are indicated at the top of the bar graphs. For retina TH levels, there were 4–6 independent replicates per treatment condition (each ethanol/caffeine dose) for a total of 35 subjects. ANOVA tables and multiple comparison results for this data can be found in S1 Table, panels (I1, I2) Retinal TH levels across pre-exposure age; (J1, J2) Retinal TH levels across time of sacrifice.

As in brain, retinal TH levels were significantly affected by exposure age, with decreased TH levels observed in older (140–149 dpf) fish (Fig 4A; p<0.008), and with increased duration of time following exposure (Fig 4B; p<0.004). This is exemplary of the dependence of neurochemical levels on age of exposure and age of sacrifice.

There was also an interaction between caffeine exposure and time of sacrifice with retinal TH showing greater levels in subjects without caffeine exposure that were sacrificed immediately, compared to those exposed to 25 mg/L caffeine and sacrificed after a long interval following exposure. We also observed significantly increased retinal TH levels overall (p<0.004) after the immediate post-exposure time interval, compared to subjects sacrificed following a long interval after exposure (Fig 4B).

#### Glutamate decarboxylase—Brain

**Fig 5:**
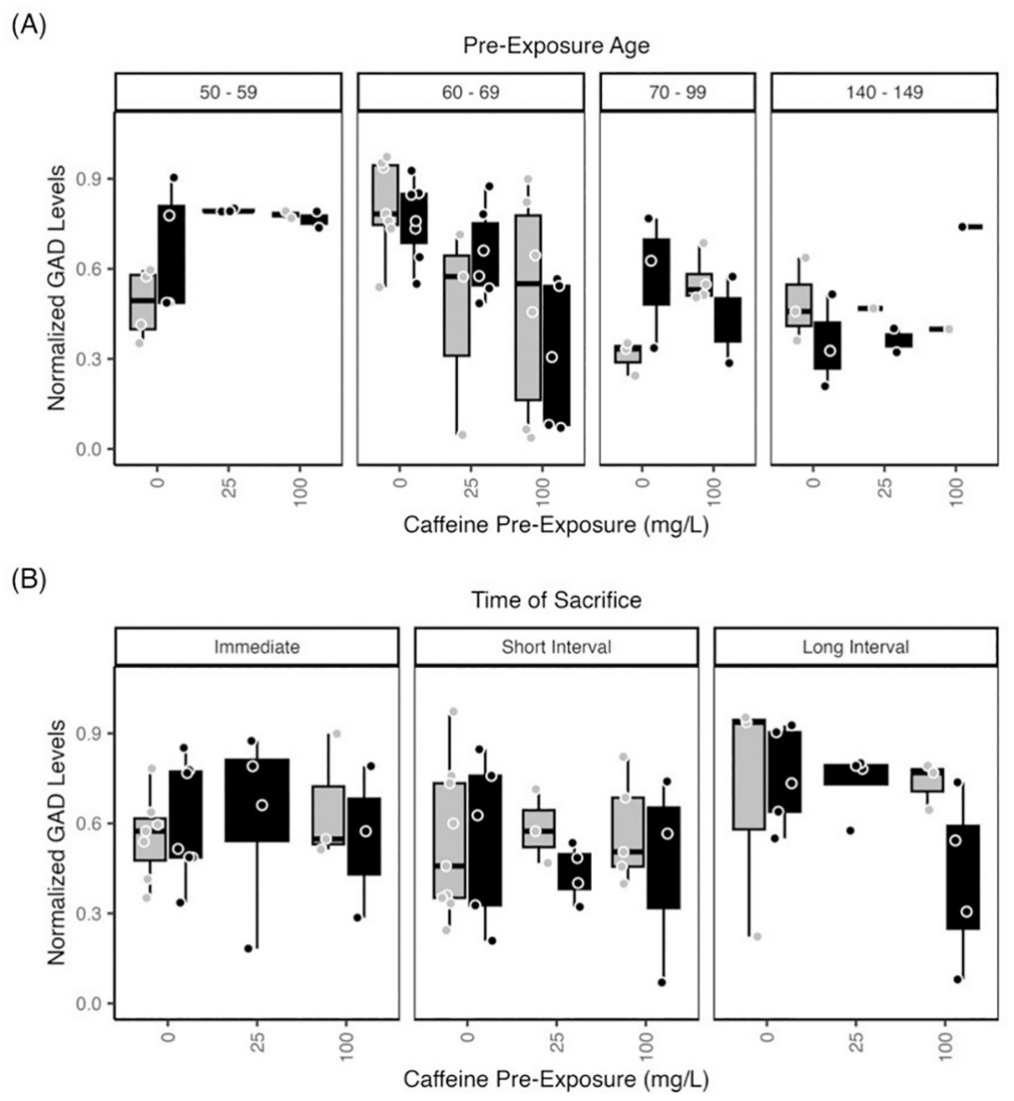
Zebrafish Brain GAD65/67 levels vary with age. Normalized brain GAD65/67 levels of zebrafish exposed to 1.5% ethanol (black bars) or water (gray bars) ± caffeine (0 mg/L; 25 mg/L, 100 mg/L). (A) There was a significant effect of pre-exposure age on brain GAD levels. GAD65/67 levels in brain tissue collected immediately after exposure showed a trend of increased levels when fish were exposed earlier, from 50–59 dpf, compared to later ages (60–149 dpf). (B) GAD65/67 levels assessed across the post-exposure time points showed a trend of greater GAD65/67 levels in subjects exposed after a long interval compared to those sacrificed immediately. The boxplots show the minimum, first quartile, median, third quartile, and maximum values for each measure after outliers were removed. Exposure ages (dpf) are indicated across the top of the bar graphs. For brain GAD levels, there were 4–6 independent replicates per treatment condition (each ethanol/caffeine dose) for a total of 35 subjects. ANOVA tables and multiple comparison results for this data can be found in S1 Table, panels (L1, L2) Brain GAD levels across pre-exposure age; (M) Brain GAD levels across time of sacrifice.

Brain GAD65/67 levels showed a significant effect of pre-exposure age (p<0.01) and a significant interaction of caffeine pre-exposure and exposure age (p<0.001) (Fig 5A). Further, brain GAD65/67 levels tended to be higher in fish exposed at 50–59 dpf compared to fish exposed at 70–99 dpf or 140–149 dpf. Brain GAD65/67 levels also increased, though not significantly, in subjects pre-exposed at 60–69 dpf compared to fish exposed at 70–99 dpf or 140–149 dpf. Using smaller age bins for the 70–99 dpf group revealed that fish exposed when they were 70–79 dpf had higher brain GAD65/67 levels than fish exposed at any other age (Fig 5A).

Notably, ethanol exposure reduced GAD65/67 levels in older subjects, but increased GAD 65/67 levels in younger subjects, an effect moderated by higher caffeine doses (Fig 5A). For example, fish exposed to either 100 mg/L or 25 mg/L caffeine showed higher levels of GAD65/67 compared to controls receiving no caffeine. Ethanol-exposed fish displayed lower levels of GAD65/67, compared to controls with no ethanol exposure.

Finally, of time of sacrifice appeared to influence brain GAD 65/67 levels (p<0.0766), with greater levels in subjects sacrificed after the long interval compared to subjects sacrificed immediately.

#### Glutamate decarboxylase—Retina

**Fig 6:**
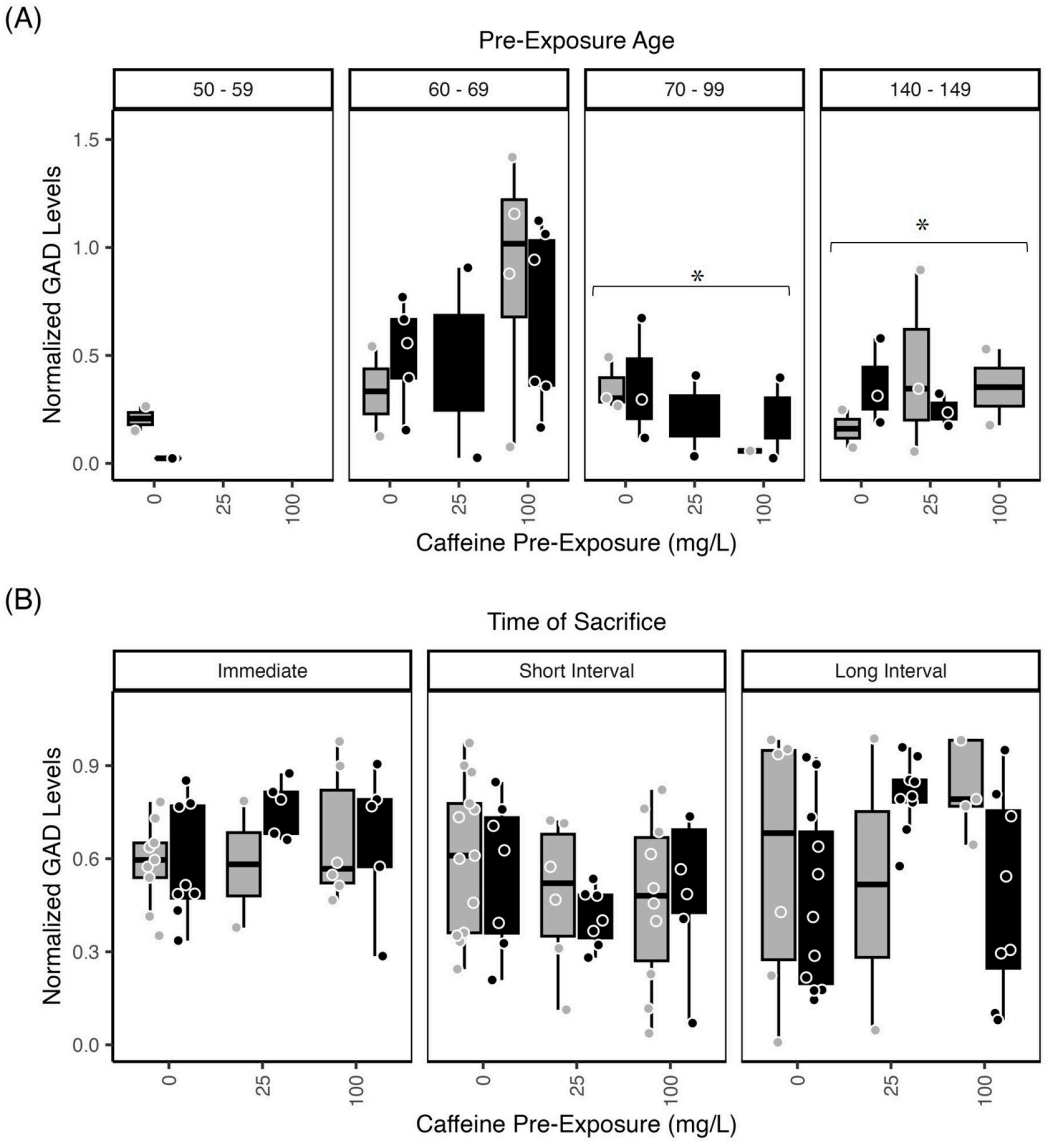
Zebrafish Retinal GAD65/67 levels were affected by caffeine. Normalized retinal GAD65/67 levels from zebrafish exposed to 1.5% ethanol (black bars) or water/control (gray bars) ± caffeine (0 mg/L; 25 mg/L, 100 mg/L). (A) GAD65/67 levels in retinal tissue measured immediately after pre-exposure were increased in fish exposed from 140–149 dpf compared to 50–69 dpf (p<0.03). Retinal GAD 65/67 levels were also increased in fish exposed from 70–99 dpf compared to 50–69 dpf (p<0.02; asterisk). (B) Retinal GAD65/67 levels assessed across the post-exposure time points did not identify any differences across groups. The boxplots show the minimum, first quartile, median, third quartile, and maximum values for each measure after outliers were removed. Exposure ages (dpf) are indicated across the top of the bar graphs. For retina GAD levels, there were 4–6 independent replicates per treatment condition (each ethanol/caffeine dose) for a total of 35 subjects. ANOVA tables and multiple comparison results for this data can be found in S1 Table, panels (N1, N2) Retinal GAD levels across pre-exposure age; and (O) Retinal GAD levels across time.

Retinal GAD65/67 levels demonstrated a significant effect of exposure age (p<0.001), with subjects exposed from 140–149 dpf showing significantly higher GAD levels than those exposed from 50–69 dpf (p<0.022) and those exposed from 70–99 dpf showing significantly higher GAD 65/67 levels than those exposed from 50–59 dpf (p<0.017) (Fig 6A). Overall, GAD65/67 levels in retina were significantly higher in older compared to younger fish.

## Discussion

The purpose of this study was to examine whether chronic 7-d exposure to ethanol and/or caffeine-induced anatomical and/or neurochemical changes, at both the whole animal and cellular level, and, if present, did the changes vary based on age of exposure (juvenile vs. adult) or timing of assessment (immediately after exposure vs. short withdrawal vs. long withdrawal + acute ethanol challenge). Our results suggest that TH and GAD65/67 levels are sensitive to ethanol and caffeine exposure in an age-dependent manner; withdrawal from exposure and/or ethanol challenge after a long (1–2 month) post-exposure interval also affected protein levels. In addition, the effect of ethanol and/or caffeine withdrawal was dependent on whether fish raised for a few days (short withdrawal) or raised for the longer period (long withdrawal) after exposure.

Neurochemical effects were observed across distinct ages of juvenile and adult fish despite a lack of impact of either drug exposure on overall morphological development. While many studies have assessed the impact of ethanol and caffeine on morphology, few have also assessed these alongside the study of neurochemical parameters. This study makes a novel contribution by assessing these neurochemical parameters in subjects with and without exposure to ethanol as well as caffeine. In addition, this study also assessed several times of sacrifice of subjects to designate the degree of persistency of the neurochemical changes observed in subjects after chronic ethanol and/or caffeine exposure in the juvenile stage. This aspect of the study was complemented by evaluation of morphological parameters to control for the overall integrity of zebrafish development despite treatment with ethanol and caffeine, which show teratogenic effects in zebrafish embryos [9].

For most our anatomical measurements, we found no significant drug-induced changes. Ethanol exposure is reported to alter zebrafish development, resulting in slower heart rate, smaller eyes, and abnormal development [34] and zebrafish exposed to caffeine display shorter body lengths [22]. A major difference of our study is age of exposure. Juveniles were the youngest age group used here, whereas embryos and larvae were used in previous studies. The juvenile stage is characterized by complete organ development, but an absence of sexual maturity [35]. In contrast, organ systems are still developing and maturing during embryonic and larval stages. This suggests early developmental sensitivity to ethanol and/or caffeine exposure. However, we did find significant age-dependent anatomical differences, with older animals having greater weight, length, eye size, and pigmentation than younger animals. Since zebrafish are known to grow as they develop through the juvenile and adult stages, these results confirm previous studies of the time-course of development of zebrafish. The older groups measured had the largest weight, length, and outer eye distance, though they were not the most heavily pigmented.

Younger fish exposed to ethanol had increased retinal TH levels. TH levels in brain were also increased in tissue assessed after the longer post-exposure time interval, relative to fish sacrificed at the earlier time points. For example, increased brain TH levels were maintained in fish 9–60 d after exposure ended and especially in tissue collected after the long post-exposure time window in the fish exposed to the high caffeine dose without ethanol. It is unclear whether the maintained TH levels were due to the long withdrawal period or the ethanol challenge. However, acute ethanol treatment enhanced brain TH levels in adult fish that received ethanol exposure as juveniles [36].

Other studies with zebrafish report that acute adult exposure to 0.25%, 0.5%, or 1% ethanol causes dose-dependent increases in DA [37] and that increased DA levels evoked by chronic 10 d exposure to 0.5% ethanol are maintained during withdrawal [38]. Removal from chronic ethanol treatment similarly increases levels of DA and TH in strain AB zebrafish, though neurotransmitter levels are similar to controls if acute ethanol treatment is given after chronic exposure [15]. Both AB and SF zebrafish display neurochemical adaptation to chronic exposure to 0.5% ethanol characterized by diminished TH levels in response to subsequent acute treatment with 0.5% or 1% ethanol [33]. Rodent models show increased *th* mRNA levels with chronic ethanol exposure, though treatment with naltrexone (an opiate receptor antagonist), and especially naltrexone and topiramate (an anticonvulsant), restores TH gene expression and inhibits ethanol self-administration [39]. Thus, previous studies in both zebrafish and rodents suggest that chronic ethanol exposure increases TH levels, which may be reversed by either subsequent acute ethanol treatment (zebrafish) or pharmaceutical intervention that counteracts the effect of ethanol (rodents).

Brain TH levels were also increased in fish exposed to caffeine, either alone or in combination with ethanol. Chronic caffeine increases TH expression in rodents [40], consistent with the present findings. Caffeine-induced increases in TH are likely due to the downstream effect of caffeine on dopaminergic neuron activity [16,41]. The degree of Conditioned Place Preference (CPP) in adult zebrafish is predictive of changes in TH levels [36] consistent with the role of TH in regulating the reward-seeking response. Co-exposure to caffeine + ethanol was expected to further increase TH levels, compared to exposure to either substance alone. However, our data suggest a more subtle effect, with changes in TH levels resulting from ethanol and/or caffeine exposure depending upon the duration of the withdrawal/when the tissue was collected after treatment. In general, ethanol’s effect on caffeine-induced increases in TH were dependent upon age of exposure. This absence of an additive effect of ethanol and caffeine on TH levels may be due to increased individual variability in neurochemical responses [42].

Both GABA and GAD65/67 are localized in the preoptic region, ventral thalamus, and hypothalamus, and their distribution in the diencephalon establishes that GABAergic cell development is heterochronic in mammals [43,44]. Since GAD65/67 exerts a regulatory role over GABA homeostasis, as the enzyme that converts glutamate to GABA, it is thought to be more susceptible to being altered by experimental treatments compared to GABA [45]. Along these lines, we have shown that brain GAD65/67 levels in zebrafish are altered in response to a 1% ethanol-conditioning dose [36].

Caffeine-induced increases in GAD65/67 protein levels in zebrafish brain were affected by age of exposure, with the trend of caffeine and ethanol increasing GAD65/67 at younger exposure ages and decreasing GAD65/67 at older exposure ages. In contrast, retinal GAD65/67 level trends were more additive, with higher GAD65/67 levels in older fish that received ethanol and caffeine. Brain GAD65/67 levels were also elevated in fish aged 50–69 dpf, across treatment groups, while retinal GAD 65/67 was elevated in fish aged 70–149 dpf, suggesting age-related sensitivity of GABAergic circuits.

The observed increase in brain GAD65/67 levels are likely due to ethanol and/or caffeine-induced changes in neural circuits that serve to reduce excitotoxicity. For example, an ethanol-induced release of NMDA receptors from inhibition may increase GAD67 levels, so dopamine and glutamatergic AMPA receptors are inhibited, exerting a protective action against excitotoxicity [46]. An increase in GAD65/67 levels may consequently contribute to protection from symptoms of withdrawal from ethanol, which otherwise is capable of inducing excitotoxic neuronal damage.

## Conclusions

Zebrafish repeatedly exposed to ethanol displayed age-dependent changes in brain TH and GAD65/67 that suggest an interactive effect of caffeine co-exposure and duration of withdrawal (post-exposure) period. Fish chronically exposed to caffeine and assessed after a 1–2 month withdrawal period have brain TH and GAD65/7 levels that vary depending upon chronic ethanol exposure. These findings are consistent with the effects of ethanol and caffeine on reward-specific neural circuits of the brain. The changes in GAD65/67 levels in the brain suggests development of a compensatory mechanism to prevent hyperexcitability due to ethanol and/or caffeine exposure.

## Supporting information

S1 Fig Original Westerns. Original/raw Western blot gels used in densitometry analysis of neurochemical experiments.
(PDF)

S2 Fig Representative Western blots of brain and retinal tissue, GAPDH boxplots, and GAPDH ANOVA tables. (A) Samples of brain (left) and retinal (right) homogenates. Exposure groups are indicated as ‘C’ = control or ‘T’ = treated. Ethanol exposure is indicated as ‘C’ = control (0% ethanol) or ‘T’ = 1.5% ethanol. Caffeine exposure is indicated as ‘C’ for control (0mg/L caffeine) or ‘T’ for 25, 50, 75, or 100mg/L caffeine exposure. GAPDH was the loading control. Density of each TH (tyrosine hydroxylase) and GAD65/67 band was normalized to the corresponding GAPDH band prior to statistical analysis. (B-E) The boxplots show the minimum, first quartile, median, third quartile, and maximum values for each measure after outliers were removed. Exposure ages (dpf) or times of sacrifice are indicated at the top of the bar graphs. (B-C) Brain GAPDH across pre-exposure ages and times of sacrifice. (F1-4) Corresponding ANOVA tables for brain tissue showing no significant differences in GAPDH levels were detected by 1-way ANOVAs for the variables of interest: Ethanol doses, caffeine doses, pre-exposure age, and time of sacrifice. (D-E) Retina GAPDH across pre-exposure ages and times of sacrifice. (F5-8) Corresponding ANOVA tables for retinal tissue showing no significant differences by 1-way ANOVAs for the variables of interest: Ethanol doses, caffeine doses, and pre-exposure age. However, the long interval for time of sacrifice (E) showed lower GAPDH levels for retina GAPDH (F8), compared to immediate and short intervals of sacrifice.
(TIF)

S1 Table ANOVA tables and multiple comparison / post hoc test results for morphology and neurochemical analyses. ANOVA tables and multiple comparison / post hoc test results for all morphological measures (A-F) and neurochemical measures (G-N) analyzed. (A1, A2) Weight; (B) Pigment density; (C1, C2) Dorsal length; (D1, D2) Sagittal Length; (E) Inner Eye Distance; (F1, F2) Outer Eye Distance; (G1, G2) Brain TH levels across pre-exposure age; (H1, H2) Brain TH levels across time of sacrifice; (I1, I2) Retinal TH levels across pre-exposure age; (J1, J2) Retinal TH levels across time of sacrifice; (K1, K2, K3) Brain GAD levels across pre-exposure age; (L) Brain GAD levels across time of sacrifice; (M1, M2) Retinal GAD levels across pre-exposure age; and (N) Retinal GAD levels across time of sacrifice. The type of ANOVA utilized is indicated in the title for each ANOVA table. In general, a 3-way ANOVA was used; however, when ANOVA requirements were violated (i.e., non-normal data based on Shapiro-Wilk test or non-homogenous variances based on Levene’s test), an Aligned Rank Transform (ART)-ANOVA was used. A significant test result is indicated with asterisks in the ‘signif.’ column in all tables. If the ANOVA did not identify significance across treatments and interactions, no post hoc test was performed.
(DOCX)
