# Supplementary material for: Ethanol and caffeine age-dependently alter brain and retinal neurochemical levels without affecting morphology of juvenile and adult zebrafish (Danio rerio)
Source: PLoS One. 2023 Jul 5;18(7):e0286596. doi: 10.1371/journal.pone.0286596 (PMC10321635; doi:10.1371/journal.pone.0286596)
Supplement: S1 Fig — Original/raw Western blot gels used in densitometry analysis of neurochemical experiments. (PDF) [file pone.0286596.s001.pdf]

TH - 60 kDa band detected

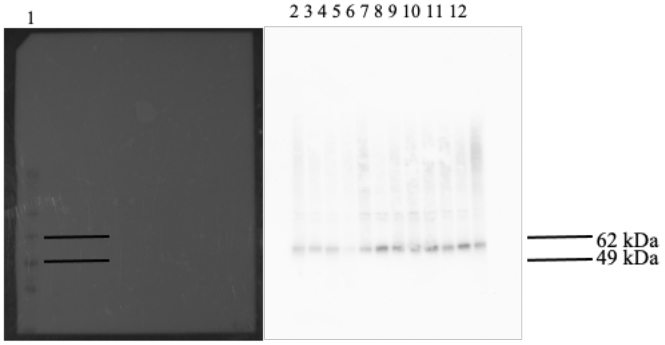

GAD 65/67 – 65/67 kDa band detected

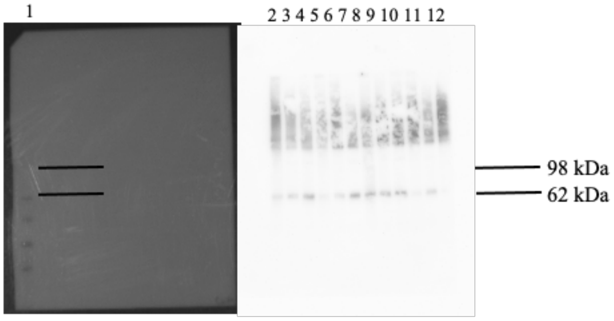

GAPDH - 36 kDa band detected

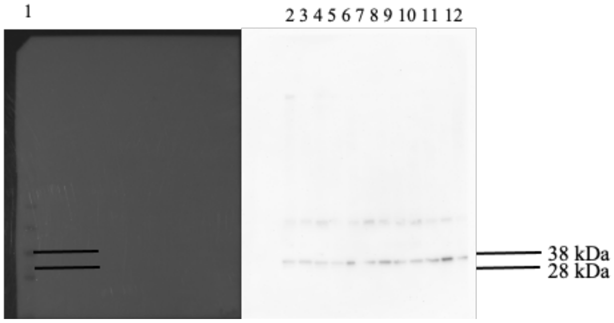

1. SeaBlue Plus2 Pre-Stained Standard
2. Brain – Control - 50-59 dpf Pre-Ex - 60-69 dpf Sacr – immediate Sacr
3. Brain – Control – 50-59 dpf Pre-Ex – 60-69 dpf Sacr – Immediate Sacr
4. Brain – 1.5% EtOH – 50-59 dpf Pre-Ex – 60-69 dpf Sacr – Immediate Sacr
5. Brain – 1.5% EtOH – 50-59 dpf Pre-Ex – 60-69 dpf Sacr – Immediate Sacr
6. Brain – 1.5% EtOH & 25 mg/L Caf – 50-59 dpf Pre-Ex – 60-69 dpf Sacr – Immediate Sacr
7. Brain – 1.5% EtOH & 25 mg/L Caf – 50-59 dpf Pre-Ex – 60-69 dpf Sacr – Immediate Sacr
8. Brain – 1.5% EtOH & 50 mg/L Caf – 50-59 dpf Pre-Ex – 60-69 dpf Sacr – Immediate Sacr
9. Brain – 1.5% EtOH & 50 mg/L Caf – 50-59 dpf Pre-Ex – 60-69 dpf Sacr – Immediate Sacr
10. Brain – 1.5% EtOH & 75 mg/L Caf – 50-59 dpf Pre-Ex – 60-69 dpf Sacr – Immediate Sacr
11. Brain – 1.5% EtOH & 75 mg/L Caf – 50-59 dpf Pre-Ex – 60-69 dpf Sacr – Immediate Sacr
12. Brain – 1.5% EtOH & 100 mg/L Caf – 50-59 dpf Pre-Ex – 60-69 dpf Sacr – Immediate Sacr

TH - 60 kDa band detected

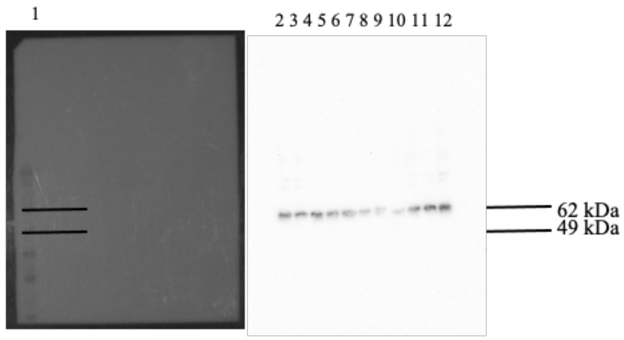

GAD 65/67 – 65/67 kDa band detected

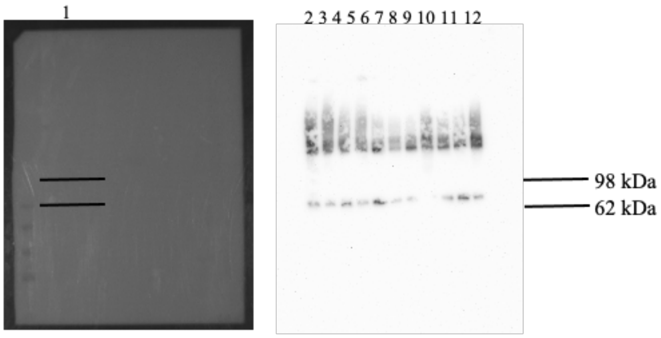

GAPDH - 36 kDa band detected

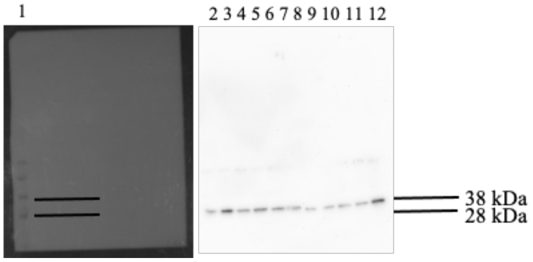

1. SeaBlue Plus2 Pre-Stained Standard
2. Brain – Control - 50-59 dpf Pre-Ex - 60-69 dpf Sacr – immediate Sacr
3. Brain – Control – 50-59 dpf Pre-Ex – 60-69 dpf Sacr – Immediate Sacr
4. Brain – Control – 50-59 dpf Pre-Ex – 60-69 dpf Sacr – Immediate Sacr
5. Brain – 1.5% EtOH – 50-59 dpf Pre-Ex – 60-69 dpf Sacr – Immediate Sacr
6. Brain – 1.5% EtOH – 50-59 dpf Pre-Ex – 60-69 dpf Sacr – Immediate Sacr
7. Brain – 1.5% EtOH & 25 mg/L Caf – 50-59 dpf Pre-Ex – 60-69 dpf Sacr – Immediate Sacr
8. Brain – 1.5% EtOH & 25 mg/L Caf – 50-59 dpf Pre-Ex – 60-69 dpf Sacr – Immediate Sacr
9. Brain – 1.5% EtOH & 50 mg/L Caf – 50-59 dpf Pre-Ex – 60-69 dpf Sacr – Immediate Sacr
10. Brain – 1.5% EtOH & 75 mg/L Caf – 50-59 dpf Pre-Ex – 60-69 dpf Sacr – Immediate Sacr
11. Brain – 1.5% EtOH & 75 mg/L Caf – 50-59 dpf Pre-Ex – 60-69 dpf Sacr – Immediate Sacr
12. Brain – 1.5% EtOH & 100 mg/L Caf – 50-59 dpf Pre-Ex – 60-69 dpf Sacr – Immediate Sacr

TH - 60 kDa band detected

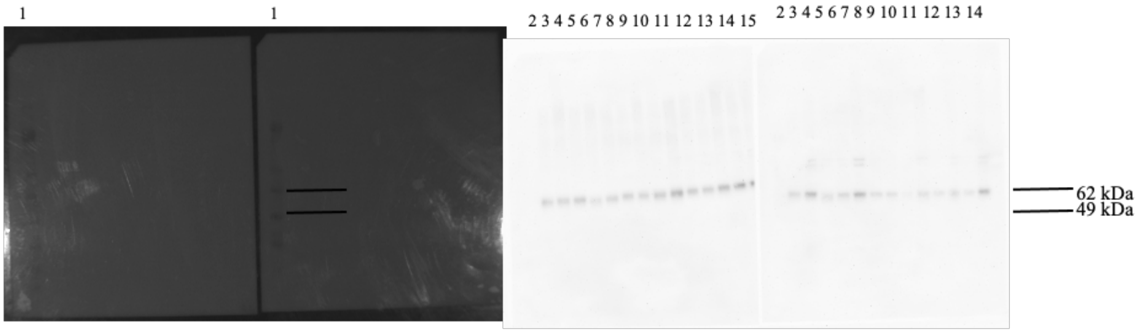

GAD 65/67 – 65/67 kDa band detected

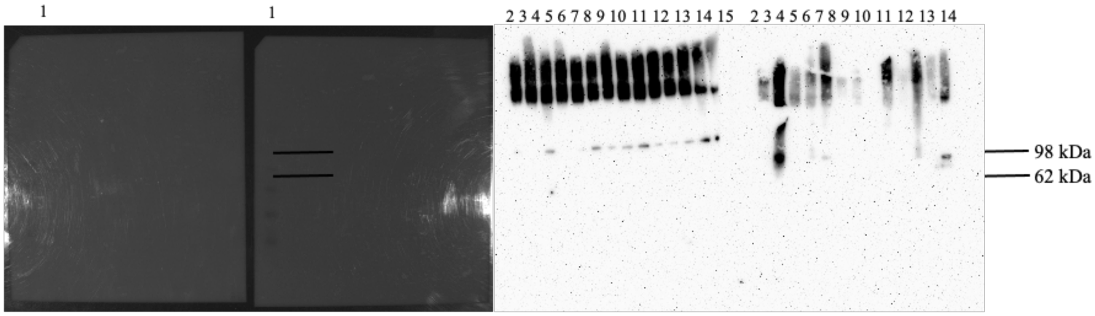

GAPDH - 36 kDa band detected

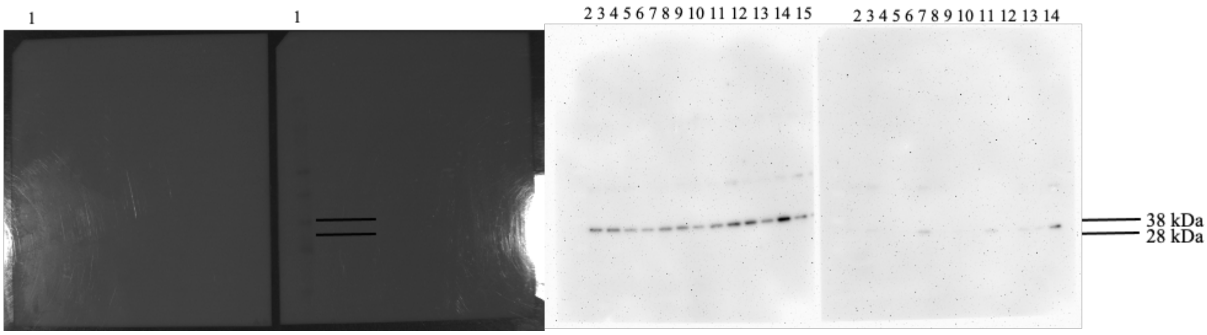

Membrane on Left

1. SeaBlue Plus2 Pre-Stained Standard
2. Brain – Control – 60-69 dpf Pre-Ex – 130-139 dpf Sacr – Long Interval Sacr
3. Brain – Control – 60-69 dpf Pre-Ex – 130-139 dpf Sacr – Long Interval Sacr
4. Brain – Control – 60-69 dpf Pre-Ex – 130-139 dpf Sacr – Long Interval Sacr
5. Brain – 1.5% EtOH – 60-69 dpf Pre-Ex – 130-139 dpf Sacr – Long Interval Sacr
6. Brain – 1.5% EtOH – 60-69 dpf Pre-Ex – 130-139 dpf Sacr – Long Interval Sacr
7. Brain – 1.5% EtOH – 60-69 dpf Pre-Ex – 130-139 dpf Sacr – Long Interval Sacr
8. Brain – 1.5% EtOH – 60-69 dpf Pre-Ex – 130-139 dpf Sacr – Long Interval Sacr
9. Brain – 1.5% EtOH – 60-69 dpf Pre-Ex – 130-139 dpf Sacr – Long Interval Sacr
10. Brain – 1.5% EtOH & 25 mg/L Caf – 60-69 dpf Pre-Ex – 130-139 dpf Sacr – Long Interval Sacr
11. Brain – 1.5% EtOH & 25 mg/L Caf – 60-69 dpf Pre-Ex – 130-139 dpf Sacr – Long Interval Sacr
12. Brain – 1.5% EtOH & 25 mg/L Caf – 60-69 dpf Pre-Ex – 130-139 dpf Sacr – Long Interval Sacr
13. Brain – 1.5% EtOH & 100 mg/L Caf – 60-69 dpf Pre-Ex – 130-139 dpf Sacr – Long Interval Sacr
14. Brain – 1.5% EtOH & 100 mg/L Caf – 60-69 dpf Pre-Ex – 130-139 dpf Sacr – Long Interval Sacr
15. Brain – 1.5% EtOH & 100 mg/L Caf – 60-69 dpf Pre-Ex – 130-139 dpf Sacr – Long Interval Sacr

Membrane on Right

1. SeaBlue Plus2 Pre-Stained Standard
2. Retina – Control – 60-69 dpf Pre-Ex – 130-139 dpf Sacr – Long Interval Sacr
3. Retina – Control – 60-69 dpf Pre-Ex – 130-139 dpf Sacr – Long Interval Sacr
4. Retina– 1.5% EtOH – 60-69 dpf Pre-Ex – 130-139 dpf Sacr – Long Interval Sacr
5. Retina – 1.5% EtOH – 60-69 dpf Pre-Ex – 130-139 dpf Sacr – Long Interval Sacr
6. Retina – 1.5% EtOH – 60-69 dpf Pre-Ex – 130-139 dpf Sacr – Long Interval Sacr
7. Retina – 1.5% EtOH – 60-69 dpf Pre-Ex – 130-139 dpf Sacr – Long Interval Sacr
8. Retina – 1.5% EtOH – 60-69 dpf Pre-Ex – 130-139 dpf Sacr – Long Interval Sacr
9. Retina – 1.5% EtOH & 25 mg/L Caf – 60-69 dpf Pre-Ex – 130-139 dpf Sacr – Long Interval Sacr
10. Retina – 1.5% EtOH & 25 mg/L Caf – 60-69 dpf Pre-Ex – 130-139 dpf Sacr – Long Interval Sacr
11. Retina – 1.5% EtOH & 100 mg/L Caf – 60-69 dpf Pre-Ex – 130-139 dpf Sacr – Long Interval Sacr
12. Retina – 1.5% EtOH & 100 mg/L Caf – 60-69 dpf Pre-Ex – 130-139 dpf Sacr – Long Interval Sacr
13. Retina – 1.5% EtOH & 100 mg/L Caf – 60-69 dpf Pre-Ex – 130-139 dpf Sacr – Long Interval Sacr
14. Retina – 1.5% EtOH & 100 mg/L Caf – 60-69 dpf Pre-Ex – 130-139 dpf Sacr – Long Interval Sacr

TH - 60 kDa band detected

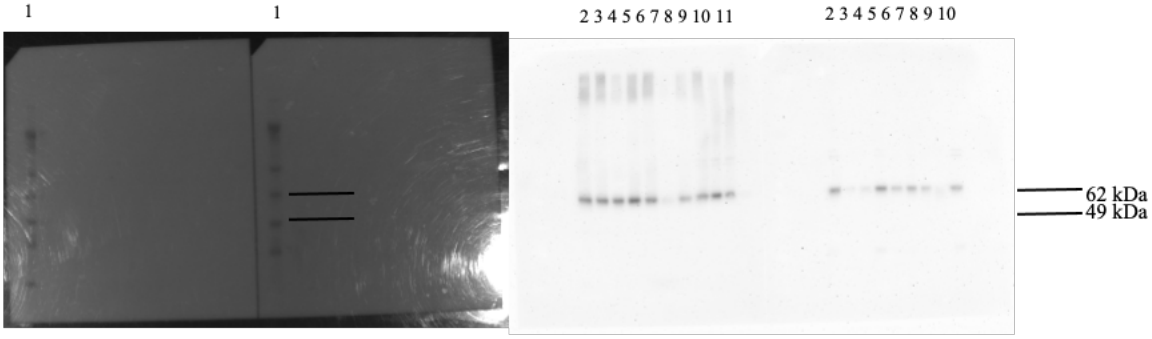

GAD 65/67 – 65/67 kDa band detected

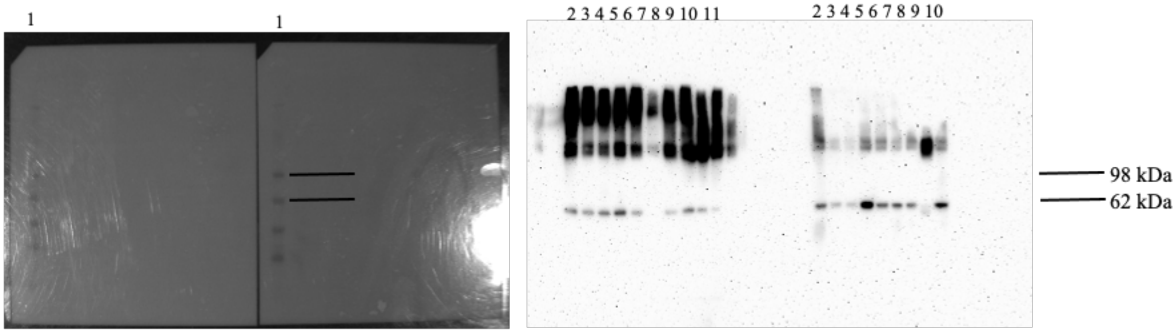

GAPDH - 36 kDa band detected

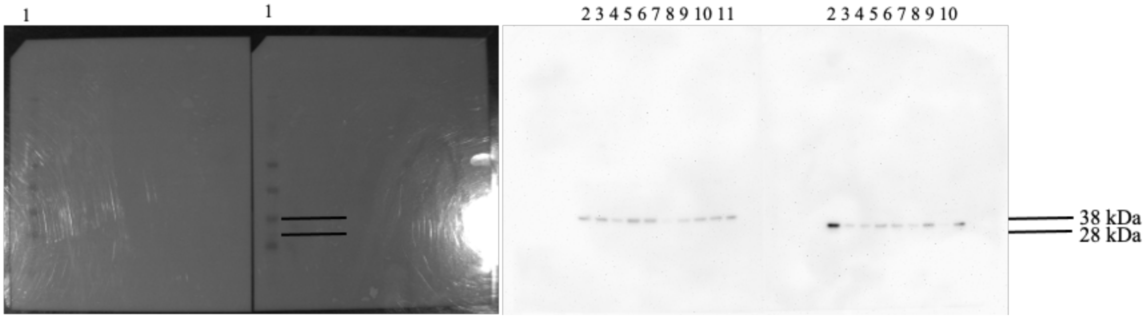

Membrane on Left
1. SeaBlue Plus2 Pre-Stained Standard
2. Brain – Control – 140-149 dpf Pre-Ex – 160-169 dpf Sacr – Short Interval Sacr
3. Brain – Control – 140-149 dpf Pre-Ex – 160-169 dpf Sacr – Short Interval Sacr
4. Brain – 1.5% EtOH– 140-149 dpf Pre-Ex – 160-169 dpf Sacr – Short Interval Sacr
5. Brain – 1.5% EtOH – 140-149 dpf Pre-Ex – 160-169 dpf Sacr – Short Interval Sacr
6. Brain – 25 mg/L Caf – 140-149 dpf Pre-Ex – 160-169 dpf Sacr – Short Interval Sacr
7. Brain – 25 mg/L Caf– 140-149 dpf Pre-Ex – 160-169 dpf Sacr – Short Interval Sacr
8. Brain – 100 mg/L Caf – 140-149 dpf Pre-Ex – 160-169 dpf Sacr – Short Interval Sacr
9. Brain – 1.5% EtOH & 25 mg/L Caf – 140-149 dpf Pre-Ex – 160-169 dpf Sacr – Short Interval Sacr
10. Brain – 1.5% EtOH & 25 mg/L Caf – 140-149 dpf Pre-Ex – 160-169 dpf Sacr – Short Interval Sacr
11. Brain – 1.5% EtOH & 100 mg/L Caf – 140-149 dpf Pre-Ex – 160-169 dpf Sacr – Short Interval Sacr

Membrane on Right
1. SeaBlue Plus2 Pre-Stained Standard
2. Retina – Control – 140-149 dpf Pre-Ex – 160-169 dpf Sacr – Short Interval Sacr
3. Retina – 1.5% EtOH – 140-149 dpf Pre-Ex – 160-169 dpf Sacr – Short Interval Sacr
4. Retina – 1.5% EtOH– 140-149 dpf Pre-Ex – 160-169 dpf Sacr – Short Interval Sacr
5. Retina – 25 mg/L Caf – 140-149 dpf Pre-Ex – 160-169 dpf Sacr – Short Interval Sacr
6. Retina – 25 mg/L Caf – 140-149 dpf Pre-Ex – 160-169 dpf Sacr – Short Interval Sacr
7. Retina – 1.5% EtOH & 25 mg/L Caf – 140-149 dpf Pre-Ex – 160-169 dpf Sacr – Short Interval Sacr
8. Retina – 1.5% EtOH & 25 mg/L Caf – 140-149 dpf Pre-Ex – 160-169 dpf Sacr – Short Interval Sacr
9. Retina – 1.5% EtOH & 100 mg/L Caf – 140-149 dpf Pre-Ex – 160-169 dpf Sacr – Short Interval Sacr
10. Retina – 1.5% EtOH & 100 mg/L Caf – 140-149 dpf Pre-Ex – 160-169 dpf Sacr – Short Interval Sacr

TH - 60 kDa band detected

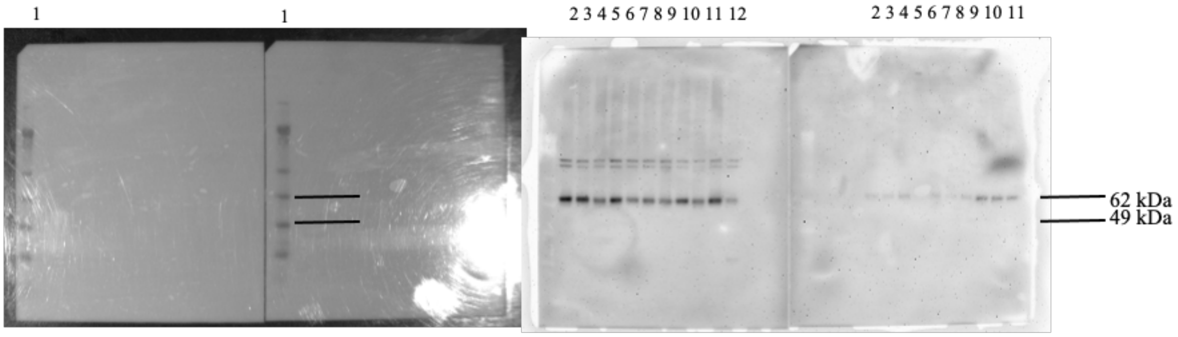

GAD 65/67 – 65/67 kDa band detected

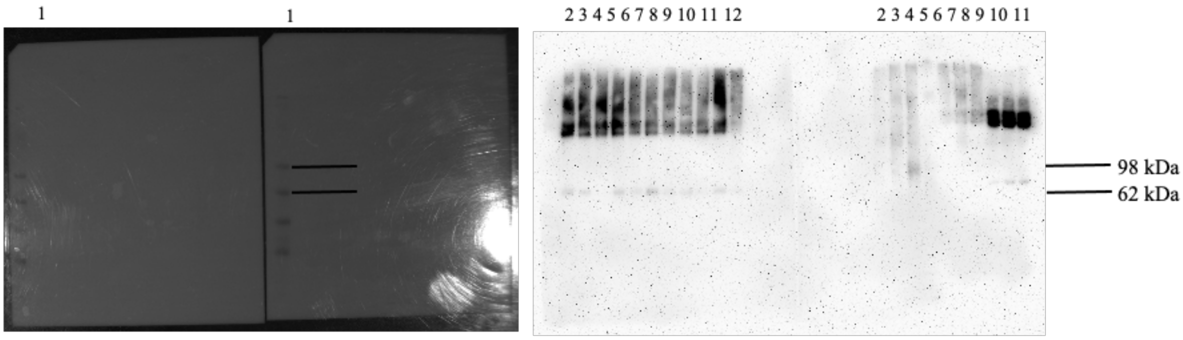

GAPDH - 36 kDa band detected

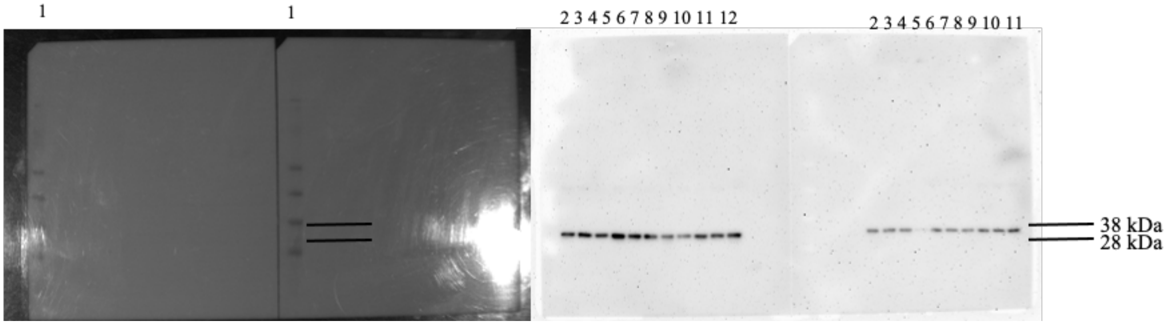

Membrane on Left

1. SeaBlue Plus2 Pre-Stained Standard
2. Brain – Control – 60-69 dpf Pre-Ex – 60-69 dpf Sacr – Immediate Sacr
3. Brain – Control – 60-69 dpf Pre-Ex – 80-89 dpf Sacr – Short Interval Sacr
4. Brain – 1.5% EtOH – 60-69 dpf Pre-Ex – 60-69 dpf Sacr – Immediate Sacr
5. Brain – 1.5% EtOH – 60-69 dpf Pre-Ex – 60-69 dpf Sacr – Short Interval Sacr
6. Brain – 1.5% EtOH – 60-69 dpf Pre-Ex – 60-69 dpf Sacr – Short Interval Sacr
7. Brain – 25 mg/L Caf – 60-69 dpf Pre-Ex – 60-69 dpf Sacr – Immediate Sacr
8. Brain – 25 mg/L Caf – 60-69 dpf Pre-Ex – 60-69 dpf Sacr – Short Interval Sacr
9. Brain – 100 mg/L Caf – 60-69 dpf Pre-Ex – 60-69 dpf Sacr – Short Interval Sacr
10. Brain – 1.5% EtOH & 25 mg/L Caf – 60-69 dpf Pre-Ex – 60-69 dpf Sacr – Immediate Sacr
11. Brain – 1.5% EtOH & 25 mg/L Caf – 60-69 dpf Pre-Ex – 60-69 dpf Sacr – Short Interval Sacr
12. Brain – 1.5% EtOH & 100 mg/L Caf – 60-69 dpf Pre-Ex – 60-69 dpf Sacr – Immediate Sacr

Membrane on Right

1. SeaBlue Plus2 Pre-Stained Standard
2. Retina – Control – 60-69 dpf Pre-Ex – 80-89 dpf Sacr – Short Interval Sacr
3. Retina – 1.5% EtOH – 60-69 dpf Pre-Ex – 80-89 dpf Sacr – Short Interval Sacr
4. Retina – 1.5% EtOH – 60-69 dpf Pre-Ex – 80-89 dpf Sacr – Short Interval Sacr
5. Retina – 25 mg/L Caf – 60-69 dpf Pre-Ex – 60-69 dpf Sacr – Immediate Sacr
6. Retina – 25 mg/L Caf – 60-69 dpf Pre-Ex – 80-89 dpf Sacr – Short Interval Sacr
7. Retina – 100 mg/L Caf – 60-69 dpf Pre-Ex – 80-89 dpf Sacr – Short Interval Sacr
8. Retina – 1.5% EtOH & 25 mg/L Caf – 60-69 dpf Pre-Ex – 80-89 dpf Sacr – Short Interval Sacr
9. Retina – 1.5% EtOH & 100 mg/L Caf – 60-69 dpf Pre-Ex – 80-89 dpf Sacr – Short Interval Sacr
10. Retina – 1.5% EtOH & 100 mg/L Caf – 60-69 dpf Pre-Ex – 80-89 dpf Sacr – Immediate Sacr
11. Retina – 1.5% EtOH & 100 mg/L Caf – 60-69 dpf Pre-Ex – 80-89 dpf Sacr – Short Interval Sacr

TH - 60 kDa band detected

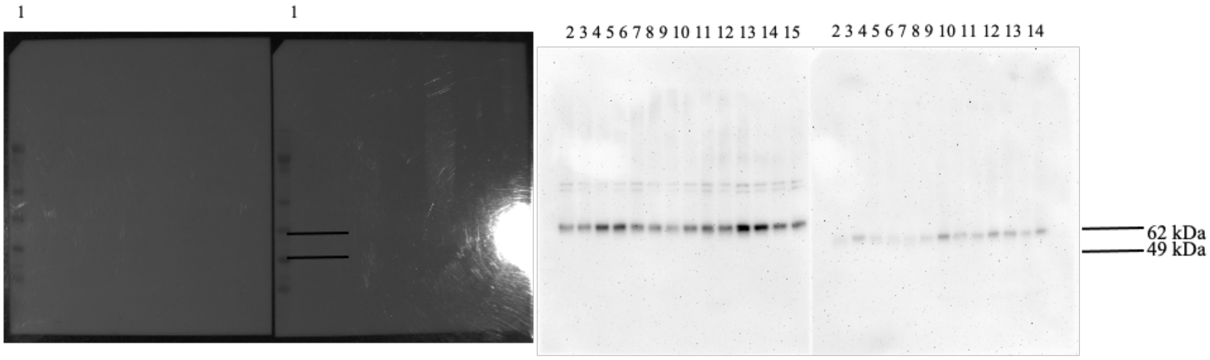

GAD 65/67 – 65/67 kDa band detected

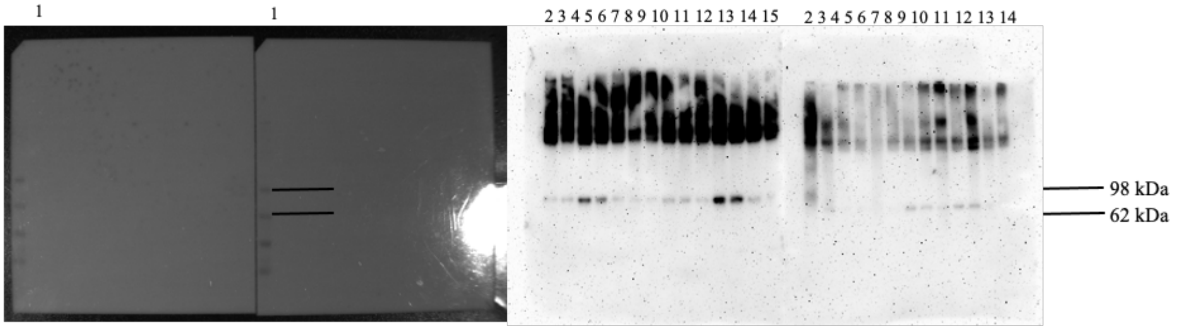

GAPDH - 36 kDa band detected

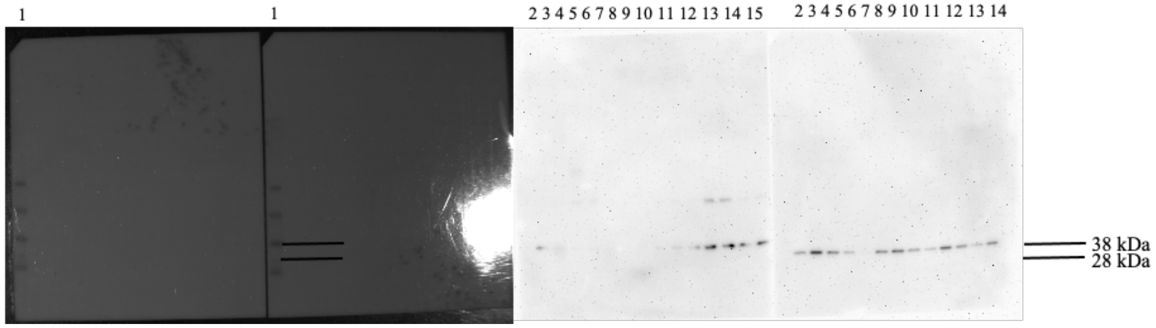

Membrane on Left
1. SeaBlue Plus2 Pre-Stained Standard
2. Brain – Control – 140-149 dpf Pre-Ex – 150-159 dpf Sacr – Immediate Sacr
3. Brain – 1.5% EtOH – 140-149 dpf Pre-Ex – 150-159 dpf Sacr – Immediate Sacr
4. Brain – 25 mg/L Caf – 140-149 dpf Pre-Ex – 150-159 dpf Sacr – Immediate Sacr
5. Brain – 100 mg/L Caf – 140-149 dpf Pre-Ex – 150-159 dpf Sacr – Immediate Sacr
6. Brain – 1.5% EtOH & 25 mg/L Caf – 140-149 dpf Pre-Ex – 150-159 dpf Sacr – Immediate Sacr
7. Brain – 1.5% EtOH & 25 mg/L Caf – 140-149 dpf Pre-Ex – 150-159 dpf Sacr – Immediate Sacr
8. Brain – 1.5% EtOH & 100 mg/L Caf – 140-149 dpf Pre-Ex – 150-159 dpf Sacr – Immediate Sacr
9. Brain – 1.5% EtOH & 100 mg/L Caf – 140-149 dpf Pre-Ex – 150-159 dpf Sacr – Immediate Sacr
10. Brain – Control– 140-149 dpf Pre-Ex – 100-109 dpf Sacr – Short Interval Sacr
11. Brain – 1.5% EtOH – 80-89 dpf Pre-Ex – 100-109 dpf Sacr – Short Interval Sacr
12. Brain – 1.5% EtOH & 25 mg/L Caf – 80-89 dpf Pre-Ex – 100-109 dpf Sacr – Short Interval Sacr
13. Brain – 1.5% EtOH & 25 mg/L Caf – 50-59 dpf Pre-Ex – 100-109 dpf Sacr – Short Interval Sacr
14. Brain – 1.5% EtOH & 25 mg/L Caf – 50-59 dpf Pre-Ex – 80-89 dpf Sacr – Long Interval Sacr
15. Brain – 1.5% EtOH & 25 mg/L Caf – 50-59 dpf Pre-Ex – 80-89 dpf Sacr – Long Interval Sacr

Membrane on Right
1. SeaBlue Plus2 Pre-Stained Standard
2. Retina – 1.5% EtOH – 80-89 dpf Pre-Ex – 90-99 dpf Sacr – Immediate Sacr
3. Retina – 1.5% EtOH – 80-89 dpf Pre-Ex – 90-99 dpf Sacr – Immediate Sacr
4. Retina – 100 mg/L Caf– 80-89 dpf Pre-Ex – 90-99 dpf Sacr – Immediate Sacr
5. Retina – 100 mg/L Caf– 80-89 dpf Pre-Ex – 90-99 dpf Sacr – Immediate Sacr
6. Retina – 1.5% EtOH & 100 mg/L Caf – 80-89 dpf Pre-Ex – 90-99 dpf Sacr – Immediate Sacr
7. Retina – Control – 140-149 dpf Pre-Ex – 150-159 dpf Sacr – Immediate Sacr
8. Retina – 1.5% EtOH – 140-149 dpf Pre-Ex – 150-159 dpf Sacr – Immediate Sacr
9. Retina – 25 mg/L Caf – 140-149 dpf Pre-Ex – 150-159 dpf Sacr – Immediate Sacr
10. Retina – 100 mg/L Caf – 140-149 dpf Pre-Ex – 150-159 dpf Sacr – Immediate Sacr
11. Retina – 1.5% EtOH & 25 mg/L Caf – 140-149 dpf Pre-Ex – 150-159 dpf Sacr – Immediate Sacr
12. Retina – 1.5% EtOH & 25 mg/L Caf – 140-149 dpf Pre-Ex – 150-159 dpf Sacr – Immediate Sacr
13. Retina – 1.5% EtOH & 100 mg/L Caf – 140-149 dpf Pre-Ex – 150-159 dpf Sacr – Immediate Sacr
14. Retina – 1.5% EtOH & 100 mg/L Caf – 140-149 dpf Pre-Ex – 150-159 dpf Sacr – Immediate Sacr

TH - 60 kDa band detected

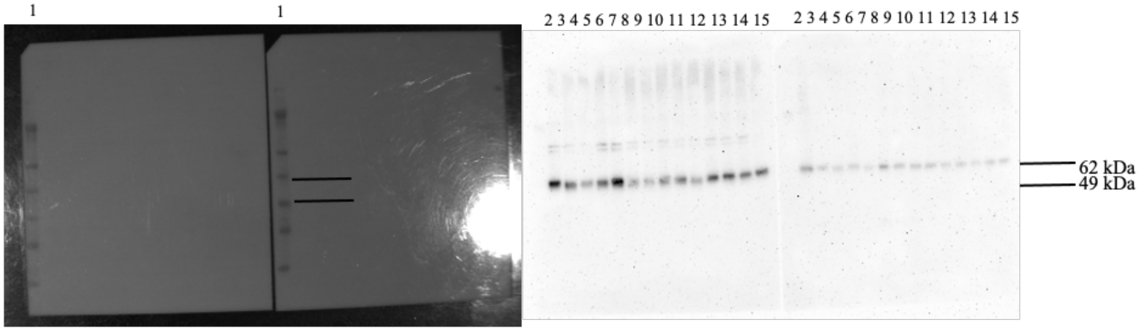

GAD 65/67 – 65/67 kDa band detected

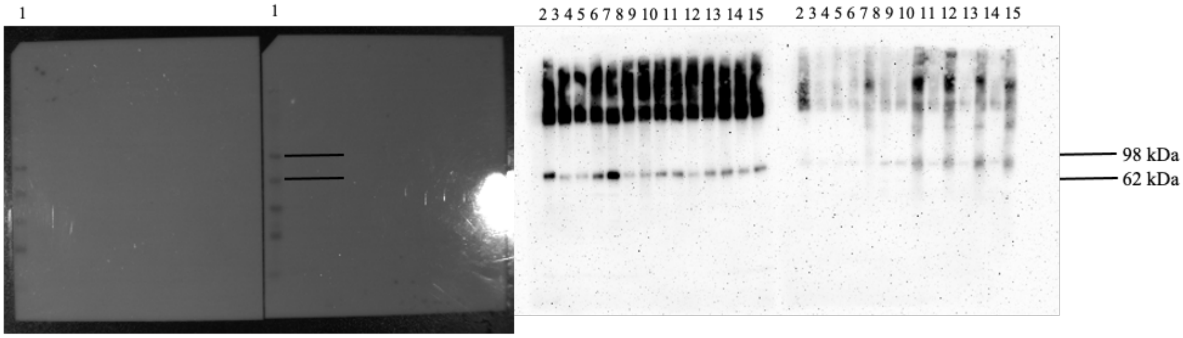

GAPDH - 36 kDa band detected

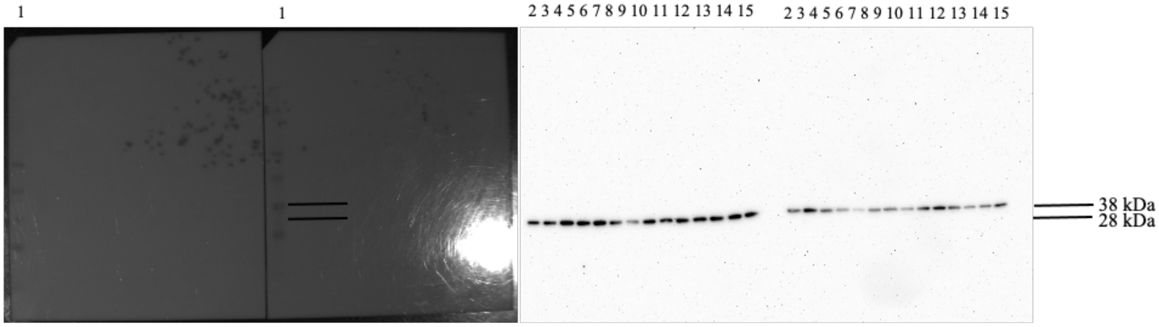

Membrane on Left

1. SeaBlue Plus2 Pre-Stained Standard
2. Brain – Control – 50-59 dpf Pre-Ex – 100-109 dpf Sacr – Long-Interval Sacr
3. Brain – 1.5% EtOH – 50-59 dpf Pre-Ex – 100-109 dpf Sacr – Long-Interval Sacr
4. Brain – 1.5% EtOH – 50-59 dpf Pre-Ex – 100-109 dpf Sacr – Long-Interval Sacr
5. Brain – 25 mg/L Caf – 50-59 dpf Pre-Ex – 100-109 dpf Sacr – Long-Interval Sacr
6. Brain – 25 mg/L Caf – 50-59 dpf Pre-Ex – 100-109 dpf Sacr – Long-Interval Sacr
7. Brain – 100 mg/L Caf – 50-59 dpf Pre-Ex – 100-109 dpf Sacr – Long-Interval Sacr
8. Brain – 100 mg/L Caf – 50-59 dpf Pre-Ex – 100-109 dpf Sacr – Long-Interval Sacr
9. Brain – 1.5% EtOH & 25 mg/L Caf – 50-59 dpf Pre-Ex – 100-109 dpf Sacr – Long-Interval Sacr
10. Brain – 1.5% EtOH & 100 mg/L Caf – 50-59 dpf Pre-Ex – 100-109 dpf Sacr – Long-Interval Sacr
11. Brain – 1.5% EtOH – 50-59 dpf Pre-Ex – 80-89 dpf Sacr – Long-Interval Sacr
12. Brain – 100 mg/L Caf – 50-59 dpf Pre-Ex – 80-89 dpf Sacr – Long-Interval Sacr
13. Brain – 100 mg/L Caf – 50-59 dpf Pre-Ex – 80-89 dpf Sacr – Long-Interval Sacr
14. Brain – 1.5% EtOH & 25 mg/L Caf – 50-59 dpf Pre-Ex – 80-89 dpf Sacr – Long-Interval Sacr
15. Brain – 1.5% EtOH & 25 mg/L Caf – 50-59 dpf Pre-Ex – 80-89 dpf Sacr – Long-Interval Sacr

Membrane on Right

1. SeaBlue Plus2 Pre-Stained Standard
2. Retina – Control – 50-59 dpf Pre-Ex – 100-109 dpf Sacr – Long-Interval Sacr
3. Retina – 1.5% EtOH – 50-59 dpf Pre-Ex – 100-109 dpf Sacr – Long-Interval Sacr
4. Retina – 25 mg/L Caf – 50-59 dpf Pre-Ex – 100-109 dpf Sacr – Long-Interval Sacr
5. Retina – 25 mg/L Caf – 50-59 dpf Pre-Ex – 100-109 dpf Sacr – Long-Interval Sacr
6. Retina – 100 mg/L Caf – 50-59 dpf Pre-Ex – 100-109 dpf Sacr – Long-Interval Sacr
7. Retina – 1.5% EtOH & 25 mg/L Caf – 50-59 dpf Pre-Ex – 100-109 dpf Sacr – Long-Interval Sacr
8. Retina – 1.5% EtOH & 100 mg/L Caf – 50-59 dpf Pre-Ex – 100-109 dpf Sacr – Long-Interval Sacr
9. Retina – 1.5% EtOH – 50-59 dpf Pre-Ex – 80-89 dpf Sacr – Long-Interval Sacr
10. Retina – 100 mg/L Caf – 50-59 dpf Pre-Ex – 80-89 dpf Sacr – Long-Interval Sacr
11. Retina – 100 mg/L Caf – 50-59 dpf Pre-Ex – 80-89 dpf Sacr – Long-Interval Sacr
12. Retina – 1.5% EtOH & 25 mg/L Caf – 50-59 dpf Pre-Ex – 80-89 dpf Sacr – Long-Interval Sacr
13. Retina – 1.5% EtOH & 25 mg/L Caf – 50-59 dpf Pre-Ex – 80-89 dpf Sacr – Long-Interval Sacr
14. Retina – 1.5% EtOH & 25 mg/L Caf – 50-59 dpf Pre-Ex – 80-89 dpf Sacr – Long-Interval Sacr
15. Retina – 1.5% EtOH & 25 mg/L Caf – 50-59 dpf Pre-Ex – 80-89 dpf Sacr – Long-Interval Sacr

TH - 60 kDa band detected

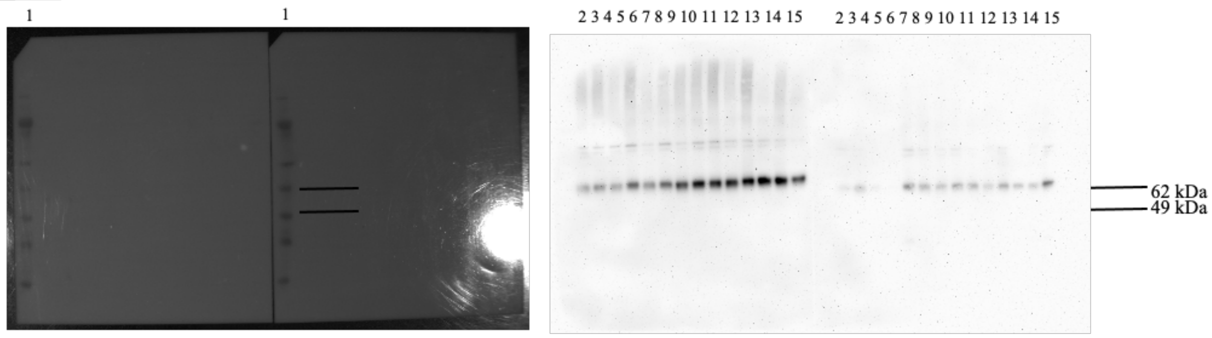

GAD 65/67 – 65/67 kDa band detected

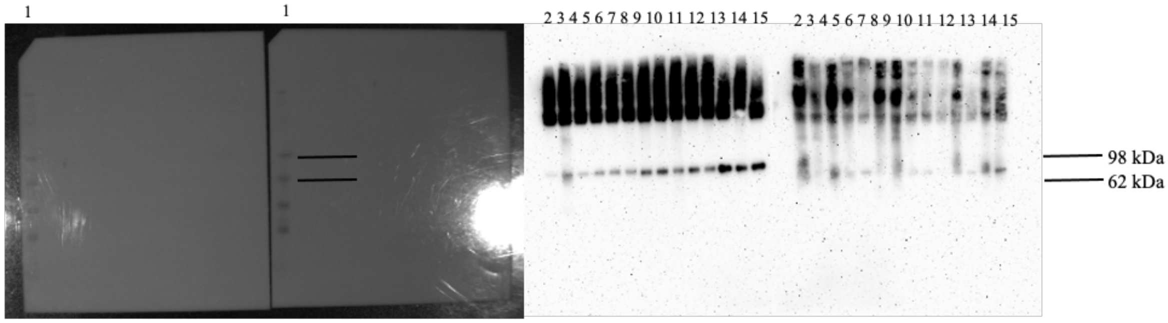

GAPDH - 36 kDa band detected

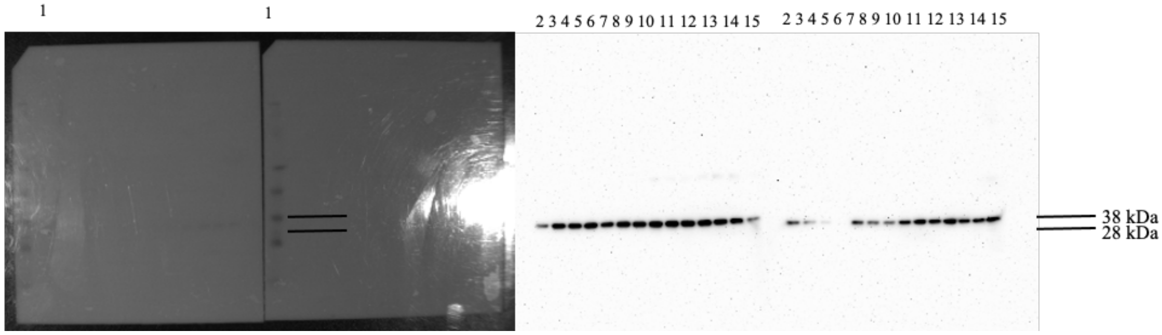

Membrane on Left

1. SeaBlue Plus2 Pre-Stained Standard
2. Brain – Control – 60-69 dpf Pre-Ex – 70-79 dpf Sacr – Immediate Sacr
3. Brain – Control – 60-69 dpf Pre-Ex – 70-79 dpf Sacr – Immediate Sacr
4. Brain – 25 mg/L Caf – 60-69 dpf Pre-Ex – 70-79 dpf Sacr – Immediate Sacr
5. Brain – 100 mg/L Caf – 60-69 dpf Pre-Ex – 70-79 dpf Sacr – Immediate Sacr
6. Brain – 1.5% EtOH & 25 mg/L Caf – 60-69 dpf Pre-Ex – 70-79 dpf Sacr – Immediate Sacr
7. Brain – 1.5% EtOH & 25 mg/L Caf – 60-69 dpf Pre-Ex – 70-79 dpf Sacr – Immediate Sacr
8. Brain – Control– 60-69 dpf Pre-Ex – 80-89 dpf Sacr – Short-Interval Sacr
9. Brain – 1.5% EtOH – 60-69 dpf Pre-Ex – 80-89 dpf Sacr – Short-Interval Sacr
10. Brain – 100 mg/L Caf – 60-69 dpf Pre-Ex – 80-89 dpf Sacr – Short-Interval Sacr
11. Brain – 1.5% EtOH & 100 mg/L Caf – 60-69 dpf Pre-Ex – 80-89 dpf Sacr – Short-Interval Sacr
12. Brain – Control – 60-69 dpf Pre-Ex – 110-119 dpf Sacr – Long-Interval Sacr
13. Brain – 1.5% EtOH – 60-69 dpf Pre-Ex – 110-119 dpf Sacr – Long-Interval Sacr
14. Brain – 100 mg/L Caf – 60-69 dpf Pre-Ex – 110-119 dpf Sacr – Long-Interval Sacr
15. Brain – 1.5% EtOH & 100 mg/L Caf – 60-69 dpf Pre-Ex – 110-119 dpf Sacr – Long-Interval Sacr

Membrane on Right

1. SeaBlue Plus2 Pre-Stained Standard
2. Retina – 100 mg/L Caf – 60-69 dpf Pre-Ex – 70-79 dpf Sacr – Immediate Sacr
3. Retina – 1.5% EtOH & 25 mg/L Caf – 60-69 dpf Pre-Ex – 70-79 dpf Sacr – Immediate Sacr
4. Retina – 1.5% EtOH & 100 mg/L Caf – 60-69 dpf Pre-Ex – 70-79 dpf Sacr – Immediate Sacr
5. Retina – Control – 60-69 dpf Pre-Ex – 80-89 dpf Sacr – Immediate Sacr
6. Retina – Control – 60-69 dpf Pre-Ex – 80-89 dpf Sacr – Immediate Sacr
7. Retina – 1.5% EtOH – 60-69 dpf Pre-Ex – 80-89 dpf Sacr – Immediate Sacr
8. Retina – 100 mg/L Caf – 60-69 dpf Pre-Ex – 80-89 dpf Sacr – Short-Interval Sacr
9. Retina – 1.5% EtOH & 25 mg/L Caf– 60-69 dpf Pre-Ex – 80-89 dpf Sacr – Short-Interval Sacr
10. Retina – 1.5% EtOH & 100 mg/L Caf – 60-69 dpf Pre-Ex – 80-89 dpf Sacr – Short-Interval Sacr
11. Retina – Control – 60-69 dpf Pre-Ex – 110-119 dpf Sacr – Long-Interval Sacr
12. Retina – 25 mg/L Caf – 60-69 dpf Pre-Ex – 110-119 dpf Sacr – Long-Interval Sacr
13. Retina – 100 mg/L Caf– 60-69 dpf Pre-Ex – 110-119 dpf Sacr – Long-Interval Sacr
14. Retina – 1.5% EtOH & 25 mg/L Caf – 60-69 dpf Pre-Ex – 110-119 dpf Sacr – Long-Interval Sacr
15. Retina – 1.5% EtOH & 100 mg/L Caf – 60-69 dpf Pre-Ex – 110-119 dpf Sacr – Long-Interval Sacr

TH - 60 kDa band detected

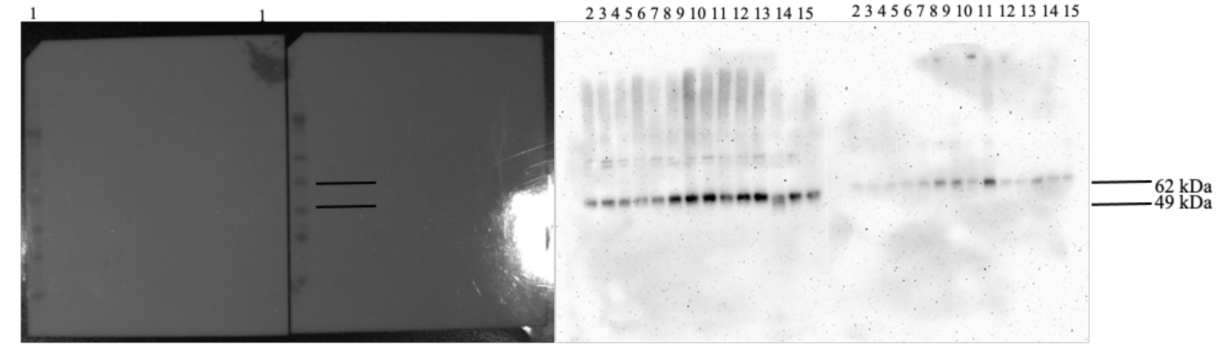

GAD 65/67 – 65/67 kDa band detected

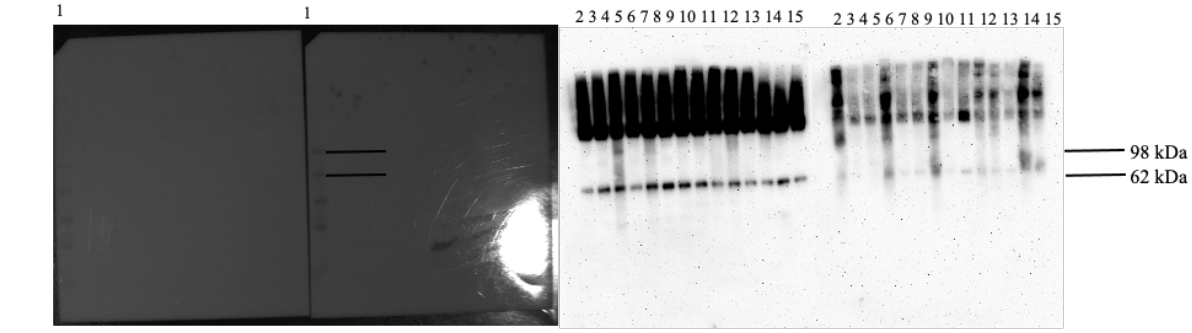

GAPDH - 36 kDa band detected

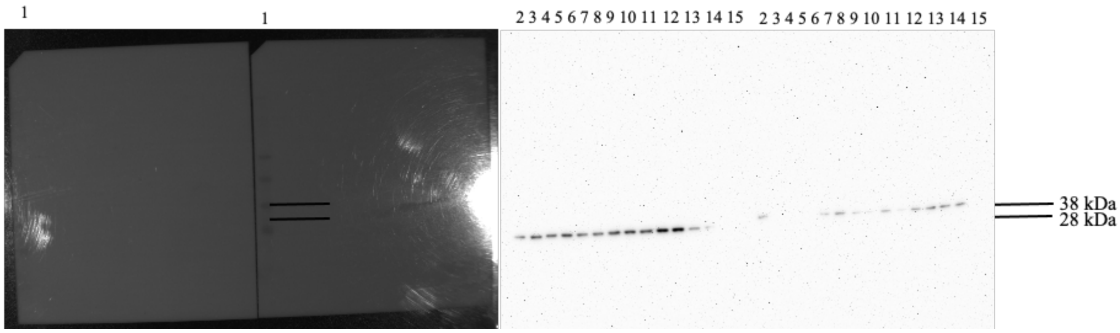

Membrane on Left

1. SeaBlue Plus2 Pre-Stained Standard
2. Brain – Control – 70-79 dpf Pre-Ex – 80-89 dpf Sacr – Immediate Sacr
3. Brain – 1.5% EtOH – 70-79 dpf Pre-Ex – 80-89 dpf Sacr – Immediate Sacr
4. Brain – 25 mg/L Caf – 70-79 dpf Pre-Ex – 80-89 dpf Sacr – Immediate Sacr
5. Brain – 100 mg/L Caf – 70-79 dpf Pre-Ex – 80-89 dpf Sacr – Immediate Sacr
6. Brain – 1.5% EtOH & 100 mg/L Caf – 70-79 dpf Pre-Ex – 80-89 dpf Sacr – Immediate Sacr
7. Brain – Control – 70-79 dpf Pre-Ex – 90-99 dpf Sacr – Short-Interval Sacr
8. Brain – Control – 70-79 dpf Pre-Ex – 90-99 dpf Sacr – Short-Interval Sacr
9. Brain – 1.5% EtOH– 70-79 dpf Pre-Ex – 90-99 dpf Sacr – Short-Interval Sacr
10. Brain – 100 mg/L Caf – 70-79 dpf Pre-Ex – 90-99 dpf Sacr – Short-Interval Sacr
11. Brain – 100 mg/L Caf – 70-79 dpf Pre-Ex – 90-99 dpf Sacr – Short-Interval Sacr
12. Brain – 1.5% EtOH & 25 mg/L Caf – 70-79 dpf Pre-Ex – 90-99 dpf Sacr – Short-Interval Sacr
13. Brain – Control – 70-79 dpf Pre-Ex – 110-119 dpf Sacr – Long-Interval Sacr
14. Brain – 1.5% EtOH – 70-79 dpf Pre-Ex – 110-119 dpf Sacr – Long-Interval Sacr
15. Brain – 100 mg/L Caf – 70-79 dpf Pre-Ex – 100-119 dpf Sacr – Long-Interval Sacr

Membrane on Right

1. SeaBlue Plus2 Pre-Stained Standard
2. Retina – Control – 70-79 dpf Pre-Ex – 80-89 dpf Sacr – Immediate Sacr
3. Retina – 100 mg/L Caf – 70-79 dpf Pre-Ex – 80-89 dpf Sacr – Immediate Sacr
4. Retina – 1.5% EtOH & 25 mg/L Caf – 70-79 dpf Pre-Ex – 80-89 dpf Sacr – Immediate Sacr
5. Retina – 1.5% EtOH & 100 mg/L Caf – 70-79 dpf Pre-Ex – 80-89 dpf Sacr – Immediate Sacr
6. Retina – Control – 60-69 dpf Pre-Ex – 90-99 dpf Sacr – Short-Interval Sacr
7. Retina – Control – 70-79 dpf Pre-Ex – 100-109 dpf Sacr – Long-Interval Sacr
8. Retina – 1.5% EtOH – 70-79 dpf Pre-Ex – 100-109 dpf Sacr – Long-Interval Sacr
9. Retina – 100 mg/L Caf – 70-79 dpf Pre-Ex – 100-109 dpf Sacr – Long-Interval Sacr
10. Retina – 1.5% EtOH & 25 mg/L Caf – 70-79 dpf Pre-Ex – 90-99 dpf Sacr – Short-Interval Sacr
11. Retina – 1.5% EtOH & 100 mg/L Caf – 90-99 dpf Pre-Ex – 100-109 dpf Sacr – Short-Interval Sacr
12. Retina – 1.5% EtOH – 60-69 dpf Pre-Ex – 80-89 dpf Sacr – Short-Interval Sacr
13. Retina – 25 mg/L Caf – 60-69 dpf Pre-Ex – 80-89 dpf Sacr – Short-Interval Sacr
14. Retina – 100 mg/L Caf – 60-69 dpf Pre-Ex – 100-109 dpf Sacr – Long-Interval Sacr
15. Retina – 1.5% EtOH & 100 mg/L Caf – 60-69 dpf Pre-Ex – 100-109 dpf Sacr – Long-Interval Sacr

TH - 60 kDa band detected

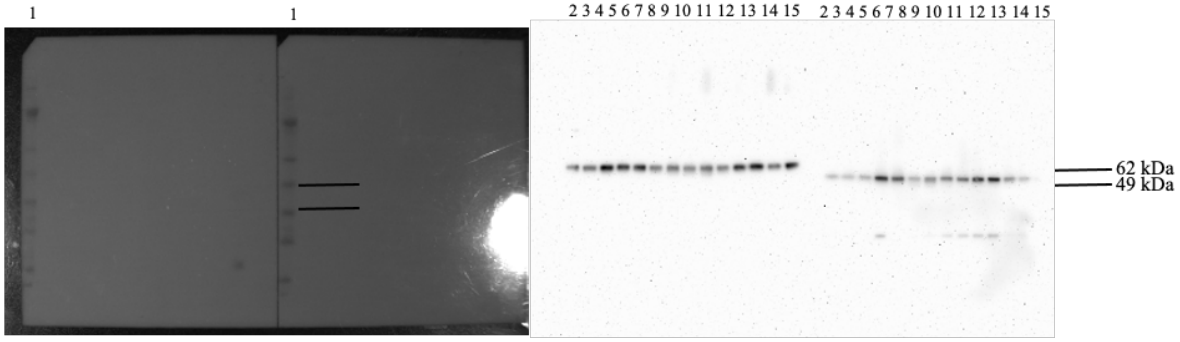

GAD 65/67 – 65/67 kDa band detected

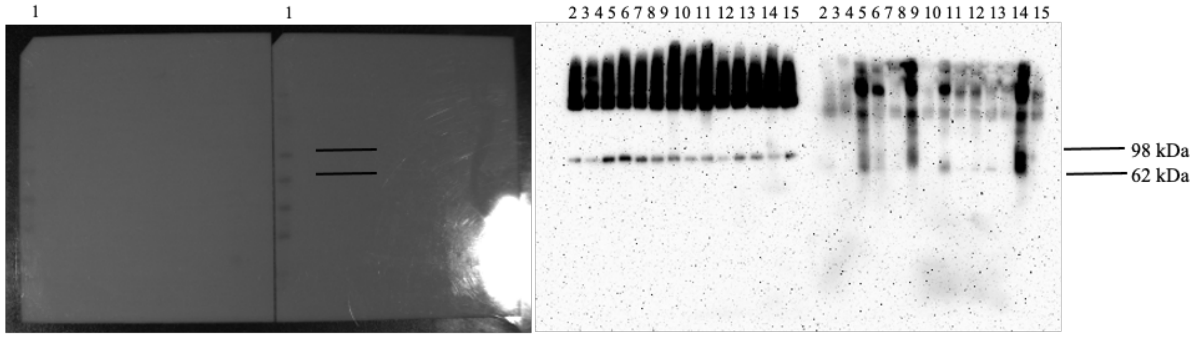

GAPDH - 36 kDa band detected

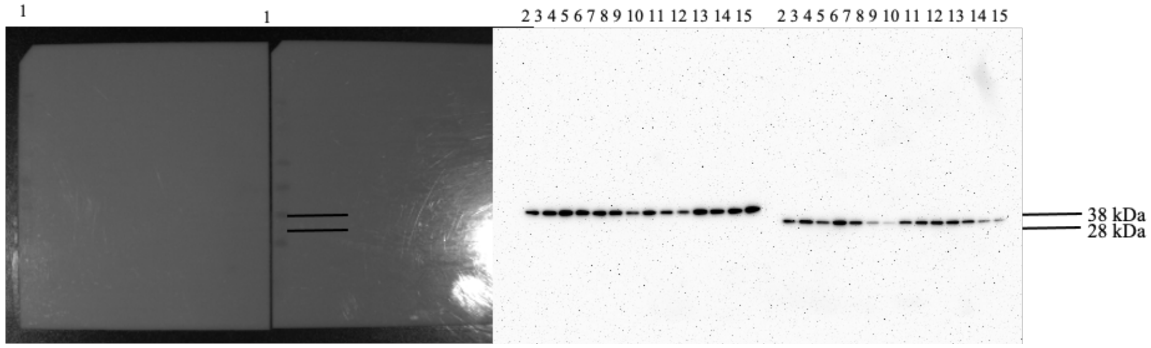

Membrane on Left

1. SeaBlue Plus2 Pre-Stained Standard
2. Brain – Control – 80-89 dpf Pre-Ex – 90-99 dpf Sacr – Immediate Sacr
3. Brain – 1.5% EtOH – 90-99 dpf Pre-Ex – 100-109 dpf Sacr – Immediate Sacr
4. Brain – 1.5% EtOH – 80-89 dpf Pre-Ex – 90-99 dpf Sacr – Immediate Sacr
5. Brain – 100 mg/L Caf – 80-89 dpf Pre-Ex – 90-99 dpf Sacr – Immediate Sacr
6. Brain – 1.5% EtOH & 100 mg/L Caf – 80-89 dpf Pre-Ex – 90-99 dpf Sacr – Immediate Sacr
7. Brain – Control – 90-99 dpf Pre-Ex – 110-119 dpf Sacr – Short-Interval Sacr
8. Brain – 1.5% EtOH – 90-99 dpf Pre-Ex – 110-119 dpf Sacr – Short-Interval Sacr
9. Brain – 100 mg/L Caf – 90-99 dpf Pre-Ex – 110-119 dpf Sacr – Short-Interval Sacr
10. Brain – 1.5% EtOH & 100 mg/L Caf– 90-99 dpf Pre-Ex – 110-119 dpf Sacr – Short-Interval Sacr
11. Brain – 1.5% EtOH & 100 mg/L Caf – 80-89 dpf Pre-Ex – 110-119 dpf Sacr – Long-Interval Sacr
12. Brain – 25 mg/L Caf – 60-69 dpf Pre-Ex – 110-119 dpf Sacr – Long-Interval Sacr
13. Brain – 100 mg/L Caf – 60-69 dpf Pre-Ex – 110-119 dpf Sacr – Long-Interval Sacr
14. Brain – 1.5% EtOH & 25 mg/L Caf – 60-69 dpf Pre-Ex – 110-119 dpf Sacr – Long-Interval Sacr
15. Brain – 1.5% EtOH & 100 mg/L Caf – 60-69 dpf Pre-Ex – 110-119 dpf Sacr – Long-Interval Sacr

Membrane on Right

1. SeaBlue Plus2 Pre-Stained Standard
2. Retina – Control – 80-89 dpf Pre-Ex – 90-99 dpf Sacr – Immediate Sacr
3. Retina – 1.5% EtOH – 80-89 dpf Pre-Ex – 90-99 dpf Sacr – Immediate Sacr
4. Retina – 100 mg/L Caf– 80-89 dpf Pre-Ex – 90-99 dpf Sacr – Immediate Sacr
5. Retina – 1.5% EtOH & 100 mg/L Caf – 80-89 dpf Pre-Ex – 90-99 dpf Sacr – Immediate Sacr
6. Retina – Control – 90-99 dpf Pre-Ex – 110-119 dpf Sacr – Short-Interval Sacr
7. Retina – 1.5% EtOH & 25 mg/L Caf – 80-89 dpf Pre-Ex – 100-109 dpf Sacr – Short-Interval Sacr
8. Retina – 100 mg/L Caf – 90-99 dpf Pre-Ex – 110-119 dpf Sacr – Short-Interval Sacr
9. Retina – 1.5% EtOH & 100 mg/L Caf– 80-89 dpf Pre-Ex – 100-109 dpf Sacr – Short-Interval Sacr
10. Retina – Control – 60-69 dpf Pre-Ex – 80-89 dpf Sacr – Short-Interval Sacr
11. Retina – 100 mg/L Caf – 60-69 dpf Pre-Ex – 80-89 dpf Sacr – Short-Interval Sacr
12. Retina – 1.5% EtOH & 100 mg/L Caf – 60-69 dpf Pre-Ex – 80-89 dpf Sacr – Short-Interval Sacr
13. Retina – 25 mg/L Caf – 60-69 dpf Pre-Ex – 110-119 dpf Sacr – Short-Interval Sacr
14. Retina – 100 mg/L Caf – 60-69 dpf Pre-Ex – 110-119 dpf Sacr – Short-Interval Sacr
15. Retina – 1.5% EtOH & 100 mg/L Caf – 60-69 dpf Pre-Ex – 110-119 dpf Sacr – Short-Interval Sacr

TH - 60 kDa band detected

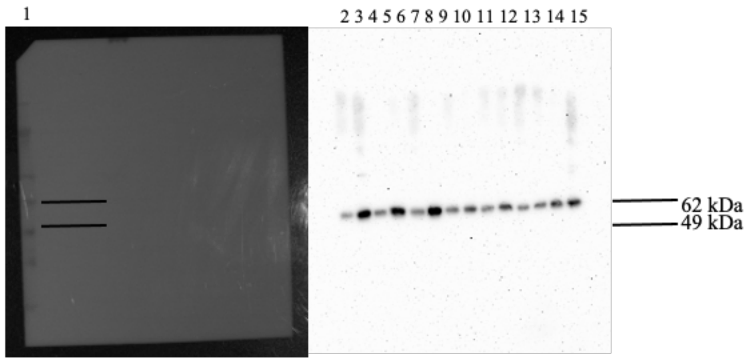

GAD 65/67 – 65/67 kDa band detected

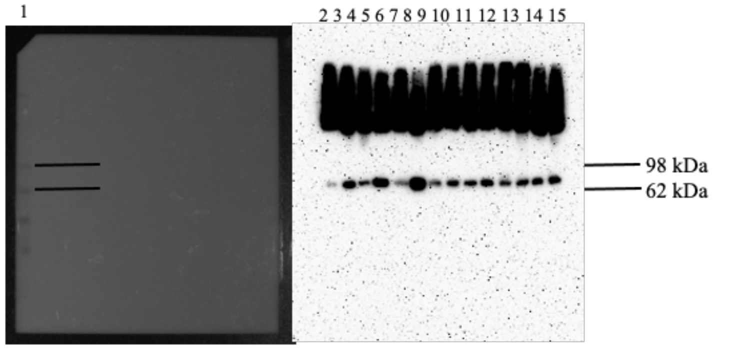

GAPDH - 36 kDa band detected

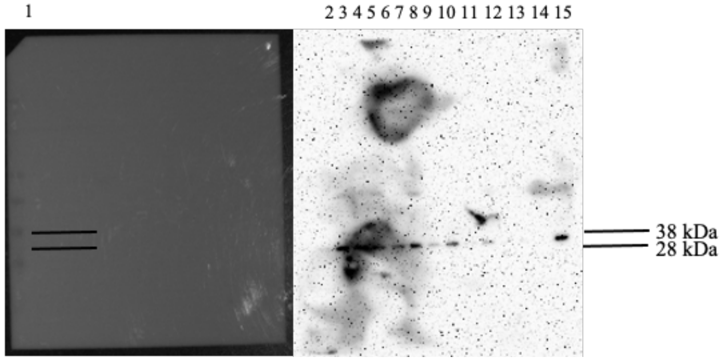

1. SeaBlue Plus2 Pre-Stained Standard
2. Brain – Control – 60-69 dpf Pre-Ex – 80-89 dpf Sacr – Short-Interval Sacr
3. Brain – Control – 60-69 dpf Pre-Ex – 80-89 dpf Sacr – Short-Interval Sacr
4. Brain – 1.5% EtOH – 60-69 dpf Pre-Ex – 80-89 dpf Sacr – Short-Interval Sacr
5. Brain – 1.5% EtOH – 60-69 dpf Pre-Ex – 80-89 dpf Sacr – Short-Interval Sacr
6. Brain – 25 mg/L Caf – 60-69 dpf Pre-Ex – 80-89 dpf Sacr – Short-Interval Sacr
7. Brain – 100 mg/L Caf – 60-69 dpf Pre-Ex – 80-89 dpf Sacr – Short-Interval Sacr
8. Brain – 1.5% EtOH & 25 mg/L Caf – 60-69 dpf Pre-Ex – 80-89 dpf Sacr – Short-Interval Sacr
9. Brain – 1.5% EtOH & 100 mg/L Caf – 60-69 dpf Pre-Ex – 80-89 dpf Sacr – Short-Interval Sacr
10. Brain – Control– 60-69 dpf Pre-Ex – 110-119 dpf Sacr – Long-Interval Sacr
11. Brain – 25 mg/L Caf – 60-69 dpf Pre-Ex – 110-119 dpf Sacr – Long-Interval Sacr
12. Brain – 100 mg/L Caf – 60-69 dpf Pre-Ex – 110-119 dpf Sacr – Long-Interval Sacr
13. Brain – 1.5% EtOH & 25 mg/L Caf – 60-69 dpf Pre-Ex – 110-119 dpf Sacr – Long-Interval Sacr
14. Brain – 1.5% EtOH & 25 mg/L Caf – 60-69 dpf Pre-Ex – 110-119 dpf Sacr – Long-Interval Sacr
15. Brain – 1.5% EtOH & 100 mg/L Caf – 60-69 dpf Pre-Ex – 110-119 dpf Sacr – Long-Interval Sacr
