# Supplementary figures and images for: Ethanol and caffeine age-dependently alter brain and retinal neurochemical levels without affecting morphology of juvenile and adult zebrafish (Danio rerio)
Source: PLoS One. 2023 Jul 5;18(7):e0286596. doi: 10.1371/journal.pone.0286596 (PMC10321635; doi:10.1371/journal.pone.0286596)

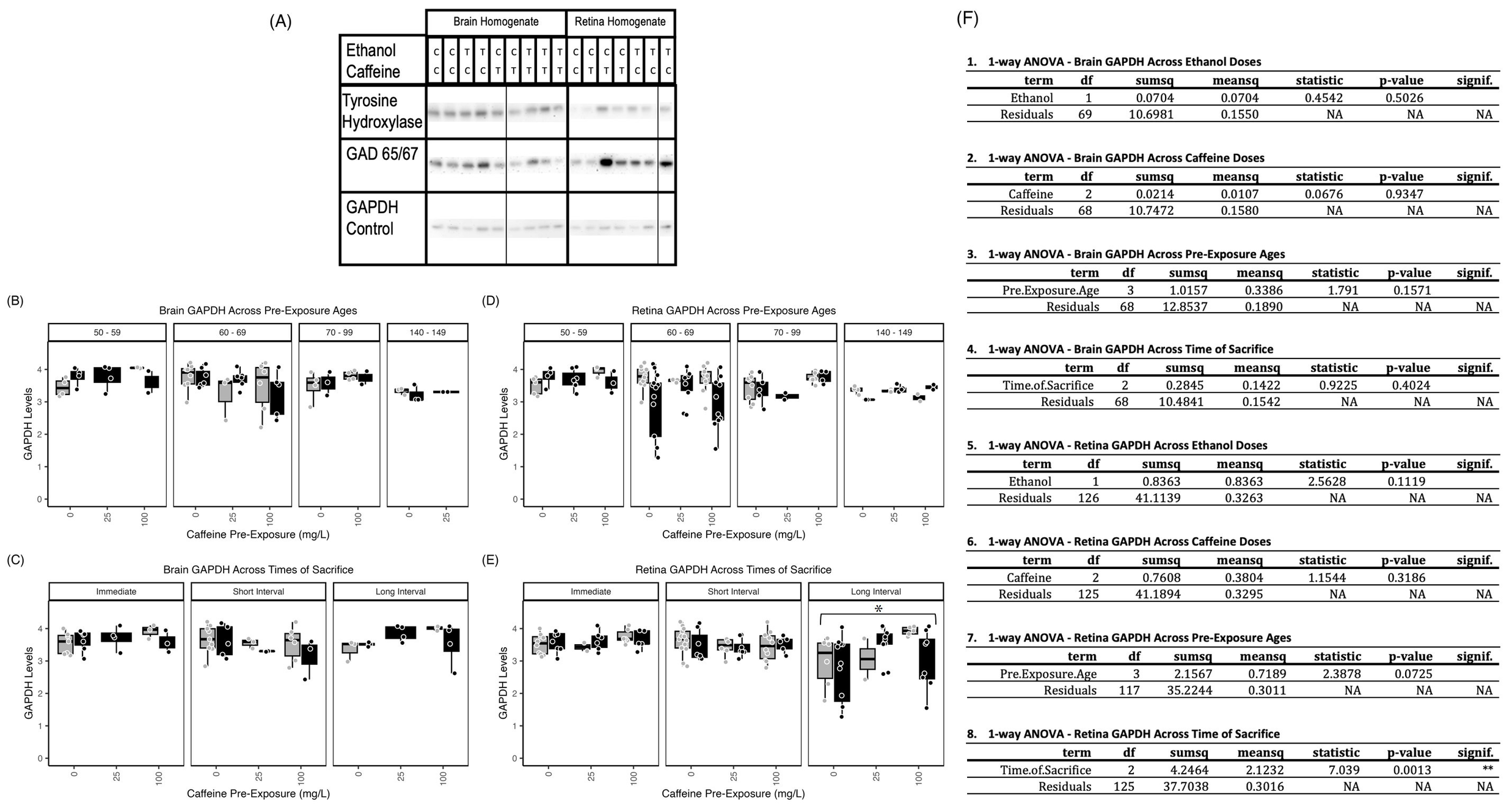

Supplement: S2 Fig — (A) Samples of brain (left) and retinal (right) homogenates. Exposure groups are indicated as ‘C’ = control or ‘T’ = treated. Ethanol exposure is indicated as ‘C’ = control (0% ethanol) or ‘T’ = 1.5% ethanol. Caffeine exposure is indicated as ‘C’ for control (0mg/L caffeine) or ‘T’ for 25, 50, 75, or 100mg/L caffeine exposure. GAPDH was the loading control. Density of each TH (tyrosine hydroxylase) and GAD65/67 band was normalized to the corresponding GAPDH band prior to statistical analysis. (B-E) The boxplots show the minimum, first quartile, median, third quartile, and maximum values for each measure after outliers were removed. Exposure ages (dpf) or times of sacrifice are indicated at the top of the bar graphs. (B-C) Brain GAPDH across pre-exposure ages and times of sacrifice. (F1-4) Corresponding ANOVA tables for brain tissue showing no significant differences in GAPDH levels were detected by 1-way ANOVAs for the variables of interest: Ethanol doses, caffeine doses, pre-exposure age, and time of sacrifice. (D-E) Retina GAPDH across pre-exposure ages and times of sacrifice. (F5-8) Corresponding ANOVA tables for retinal tissue showing no significant differences by 1-way ANOVAs for the variables of interest: Ethanol doses, caffeine doses, and pre-exposure age. However, the long interval for time of sacrifice (E) showed lower GAPDH levels for retina GAPDH (F8), compared to immediate and short intervals of sacrifice. (TIF) [file pone.0286596.s002.tif]
