# Supplementary material for: Ethanol and caffeine age-dependently alter brain and retinal neurochemical levels without affecting morphology of juvenile and adult zebrafish (Danio rerio)
Source: PLoS One. 2023 Jul 5;18(7):e0286596. doi: 10.1371/journal.pone.0286596 (PMC10321635; doi:10.1371/journal.pone.0286596)
Supplement: S1 Table — ANOVA tables and multiple comparison / post hoc test results for all morphological measures (A-F) and neurochemical measures (G-N) analyzed. (A1, A2) Weight; (B) Pigment density; (C1, C2) Dorsal length; (D1, D2) Sagittal Length; (E) Inner Eye Distance; (F1, F2) Outer Eye Distance; (G1, G2) Brain TH levels across pre-exposure age; (H1, H2) Brain TH levels across time of sacrifice; (I1, I2) Retinal TH levels across pre-exposure age; (J1, J2) Retinal TH levels across time of sacrifice; (K1, K2, K3) Brain GAD levels across pre-exposure age; (L) Brain GAD levels across time of sacrifice; (M1, M2) Retinal GAD levels across pre-exposure age; and (N) Retinal GAD levels across time of sacrifice. The type of ANOVA utilized is indicated in the title for each ANOVA table. In general, a 3-way ANOVA was used; however, when ANOVA requirements were violated (i.e., non-normal data based on Shapiro-Wilk test or non-homogenous variances based on Levene’s test), an Aligned Rank Transform (ART)-ANOVA was used. A significant test result is indicated with asterisks in the ‘signif.’ column in all tables. If the ANOVA did not identify significance across treatments and interactions, no post hoc test was performed. (DOCX) [file pone.0286596.s003.docx]

# Table S1. ANOVA tables and posthoc test results for Morphology and Neurochemical Analysis.

# (A1) ANOVA - Weight (grams)

| term | df | sumsq | meansq | statistic | p-value | signif. |
| --- | --- | --- | --- | --- | --- | --- |
| Ethanol | 1 | 0.0013 | 0.0013 | 0.2645 | 0.6093 |  |
| Caffeine | 2 | 0.0292 | 0.0146 | 3.0799 | 0.0544 |  |
| Age.of.Sacrifice | 2 | 0.5112 | 0.2556 | 53.9932 | 0.0000 | *** |
| Ethanol:Caffeine | 2 | 0.0001 | 0.0001 | 0.0106 | 0.9894 |  |
| Ethanol:Age.of.Sacrifice | 2 | 0.0049 | 0.0025 | 0.5227 | 0.5960 |  |
| Caffeine:Age.of.Sacrifice | 4 | 0.0098 | 0.0025 | 0.5188 | 0.7223 |  |
| Ethanol:Caffeine:Age.of.Sacrifice | 3 | 0.0090 | 0.0030 | 0.6304 | 0.5987 |  |
| Residuals | 52 | 0.2462 | 0.0047 | NA | NA | NA |

# (A2) Post hoc test for Age of Sacrifice - Weight (grams)

| group1 | group2 | adjusted p-value | signif. |
| --- | --- | --- | --- |
| 80 - 89 | 60 - 79 | 0.3637 |  |
| 160 - 169 | 60 - 79 | 0.0000 | *** |
| 160 - 169 | 80 - 89 | 0.0000 | *** |

# (B) ANOVA - Pigment Density

| term | df | sumsq | meansq | statistic | p-value | signif. |
| --- | --- | --- | --- | --- | --- | --- |
| Ethanol | 1 | 0.0021 | 0.0021 | 0.0142 | 0.9054 |  |
| Caffeine | 2 | 0.0148 | 0.0074 | 0.0510 | 0.9503 |  |
| Age.of.Sacrifice | 4 | 0.1640 | 0.0410 | 0.2824 | 0.8890 |  |
| Ethanol:Caffeine | 2 | 0.1538 | 0.0769 | 0.5296 | 0.5900 |  |
| Ethanol:Age.of.Sacrifice | 4 | 0.2107 | 0.0527 | 0.3627 | 0.8349 |  |
| Caffeine:Age.of.Sacrifice | 8 | 0.5455 | 0.0682 | 0.4696 | 0.8760 |  |
| Ethanol:Caffeine:Age.of.Sacrifice | 6 | 0.3511 | 0.0585 | 0.4030 | 0.8762 |  |
| Residuals | 150 | 21.7801 | 0.1452 | NA | NA | NA |

# (C1) ANOVA - Dorsal Length

| term | df | sumsq | meansq | statistic | p-value | signif. |
| --- | --- | --- | --- | --- | --- | --- |
| Ethanol | 1 | 6.3212 | 6.3212 | 1.4126 | 0.2365 |  |
| Caffeine | 2 | 0.5418 | 0.2709 | 0.0605 | 0.9413 |  |
| Age.of.Sacrifice | 2 | 289.9375 | 144.9687 | 32.3952 | 0.0000 | *** |
| Ethanol:Caffeine | 2 | 14.8570 | 7.4285 | 1.6600 | 0.1936 |  |
| Ethanol:Age.of.Sacrifice | 2 | 14.4307 | 7.2153 | 1.6124 | 0.2029 |  |
| Caffeine:Age.of.Sacrifice | 3 | 20.7442 | 6.9147 | 1.5452 | 0.2052 |  |
| Ethanol:Caffeine:Age.of.Sacrifice | 3 | 9.1206 | 3.0402 | 0.6794 | 0.5660 |  |
| Residuals | 150 | 671.2506 | 4.4750 | NA | NA | NA |

# (C2) Post hoc test for Age of Sacrifice - Dorsal Length

| group1 | group2 | adjusted p-value | signif. |
| --- | --- | --- | --- |
| 80-99 | 60-79 | 0.0752 |  |
| 100-119 | 60-79 | 0.0000 | *** |
| 100-119 | 80-99 | 0.0000 | *** |

# (D1) ANOVA - Sagittal Length

| term | Term | df | Df.res | sumsq | Sum.Sq.res | statistic | p-value | signif. |
| --- | --- | --- | --- | --- | --- | --- | --- | --- |
| Ethanol | Ethanol | 1 | 133 | 1364.3097 | 252126.7 | 0.7197 | 0.3978 |  |
| Caffeine | Caffeine | 2 | 133 | 1222.1702 | 252364.9 | 0.3221 | 0.7252 |  |
| Age.of.Sacrifice | Age.of.Sacrifice | 1 | 133 | 51397.8995 | 200354.1 | 34.1192 | 0.0000 | *** |
| Ethanol:Caffeine | Ethanol:Caffeine | 2 | 133 | 8940.3080 | 244379.6 | 2.4328 | 0.0917 |  |
| Ethanol:Age.of.Sacrifice | Ethanol:Age.of.Sacrifice | 1 | 133 | 3921.0463 | 249128.8 | 2.0933 | 0.1503 |  |
| Caffeine:Age.of.Sacrifice | Caffeine:Age.of.Sacrifice | 2 | 133 | 5165.3401 | 248587.8 | 1.3818 | 0.2547 |  |
| Ethanol:Caffeine:Age.of.Sacrifice | Ethanol:Caffeine:Age.of.Sacrifice | 2 | 133 | 470.1651 | 251561.5 | 0.1243 | 0.8832 |  |

# (D2) Post hoc test for Age of Sacrifice - Sagittal Length

| term | contrast | null.value | estimate | std.error | df | statistic | adjusted p-value | signif. |
| --- | --- | --- | --- | --- | --- | --- | --- | --- |
| Age.of.Sacrifice | (80 - 99) - (100 - 119) | 0 | -38.17589 | 6.535664 | 133 | -5.841164 | 0 | *** |

# (E) Aligned Rank Transform ANOVA - Inner Eye Distance

| term | Term | df | Df.res | sumsq | Sum.Sq.res | statistic | p-value | signif. |
| --- | --- | --- | --- | --- | --- | --- | --- | --- |
| Ethanol | Ethanol | 1 | 132 | 24.6034 | 246813.2 | 0.0132 | 0.9088 |  |
| Caffeine | Caffeine | 2 | 132 | 1319.6862 | 245788.8 | 0.3544 | 0.7023 |  |
| Age.of.Sacrifice | Age.of.Sacrifice | 1 | 132 | 2721.3318 | 245012.4 | 1.4661 | 0.2281 |  |
| Ethanol:Caffeine | Ethanol:Caffeine | 2 | 132 | 1590.4539 | 245887.7 | 0.4269 | 0.6534 |  |
| Ethanol:Age.of.Sacrifice | Ethanol:Age.of.Sacrifice | 1 | 132 | 9.3418 | 246883.6 | 0.0050 | 0.9438 |  |
| Caffeine:Age.of.Sacrifice | Caffeine:Age.of.Sacrifice | 2 | 132 | 6949.0348 | 240041.0 | 1.9107 | 0.1521 |  |
| Ethanol:Caffeine:Age.of.Sacrifice | Ethanol:Caffeine:Age.of.Sacrifice | 2 | 132 | 221.1427 | 246160.8 | 0.0593 | 0.9425 |  |

# (F1) Aligned Rank Transform ANOVA - Outer Eye Distance

| term | Term | df | Df.res | sumsq | Sum.Sq.res | statistic | p-value | signif. |
| --- | --- | --- | --- | --- | --- | --- | --- | --- |
| Ethanol | Ethanol | 1 | 130 | 857.8422 | 236778.8 | 0.4710 | 0.4938 |  |
| Caffeine | Caffeine | 2 | 130 | 1320.7886 | 236427.4 | 0.3631 | 0.6962 |  |
| Age.of.Sacrifice | Age.of.Sacrifice | 1 | 130 | 48137.3417 | 187413.8 | 33.3906 | 0.0000 | *** |
| Ethanol:Caffeine | Ethanol:Caffeine | 2 | 130 | 4236.3108 | 233217.4 | 1.1807 | 0.3103 |  |
| Ethanol:Age.of.Sacrifice | Ethanol:Age.of.Sacrifice | 1 | 130 | 2414.7118 | 234974.1 | 1.3359 | 0.2499 |  |
| Caffeine:Age.of.Sacrifice | Caffeine:Age.of.Sacrifice | 2 | 130 | 8547.6082 | 229091.4 | 2.4252 | 0.0925 |  |
| Ethanol:Caffeine:Age.of.Sacrifice | Ethanol:Caffeine:Age.of.Sacrifice | 2 | 130 | 815.5395 | 235753.9 | 0.2249 | 0.7989 |  |

# (F2) Post hoc test for Age of Sacrifice - Outer Eye Distance

| term | contrast | null.value | estimate | std.error | df | statistic | adjusted p-value | signif. |
| --- | --- | --- | --- | --- | --- | --- | --- | --- |
| Age.of.Sacrifice | (80 - 99) - (100 - 119) | 0 | -37.23303 | 6.44342 | 130 | -5.778458 | 0 | *** |

# (G1) ANOVA - Brain TH Across Pre-Exposure Ages

| term | df | sumsq | meansq | statistic | p-value | signif. |
| --- | --- | --- | --- | --- | --- | --- |
| Ethanol | 1 | 0.0000 | 0.0000 | 0.0001 | 0.9906 |  |
| Caffeine | 2 | 0.0022 | 0.0011 | 0.0060 | 0.9940 |  |
| Pre.Exposure.Age | 3 | 3.4553 | 1.1518 | 6.2239 | 0.0011 | ** |
| Ethanol:Caffeine | 2 | 0.2062 | 0.1031 | 0.5570 | 0.5762 |  |
| Ethanol:Pre.Exposure.Age | 3 | 0.2075 | 0.0692 | 0.3737 | 0.7723 |  |
| Caffeine:Pre.Exposure.Age | 4 | 1.2397 | 0.3099 | 1.6748 | 0.1696 |  |
| Ethanol:Caffeine:Pre.Exposure.Age | 2 | 0.1162 | 0.0581 | 0.3140 | 0.7318 |  |
| Residuals | 53 | 9.8078 | 0.1851 | NA | NA | NA |

# (G2) Post hoc test for Pre-Exposure Age - Brain TH Across Pre-Exposure Ages

| group1 | group2 | adjusted p-value | signif. |
| --- | --- | --- | --- |
| 60 - 69 | 50 - 59 | 0.2639 |  |
| 70 - 99 | 50 - 59 | 0.7722 |  |
| 70 - 99 | 60 - 69 | 0.7722 |  |
| 140 - 149 | 50 - 59 | 0.0005 | *** |
| 140 - 149 | 60 - 69 | 0.0059 | ** |
| 140 - 149 | 70 - 99 | 0.0044 | ** |

# (H1) ANOVA - Brain TH Across Time of Sacrifice

| term | df | sumsq | meansq | statistic | p-value | signif. |
| --- | --- | --- | --- | --- | --- | --- |
| Ethanol | 1 | 0.0055 | 0.0055 | 0.1604 | 0.6904 |  |
| Caffeine | 2 | 0.0024 | 0.0012 | 0.0344 | 0.9662 |  |
| Time.of.Sacrifice | 2 | 0.4783 | 0.2391 | 6.9301 | 0.0021 | ** |
| Ethanol:Caffeine | 2 | 0.0211 | 0.0106 | 0.3062 | 0.7375 |  |
| Ethanol:Time.of.Sacrifice | 2 | 0.0392 | 0.0196 | 0.5674 | 0.5703 |  |
| Caffeine:Time.of.Sacrifice | 4 | 0.0751 | 0.0188 | 0.5444 | 0.7038 |  |
| Ethanol:Caffeine:Time.of.Sacrifice | 2 | 0.0498 | 0.0249 | 0.7221 | 0.4904 |  |
| Residuals | 54 | 1.8633 | 0.0345 | NA | NA | NA |

# (H2) Post hoc test for Time of Sacrifice - Brain TH Across Time of Sacrifice

| group1 | group2 | adjusted p-value | signif. |
| --- | --- | --- | --- |
| Short Interval | Immediate | 0.0037 | ** |
| Long Interval | Immediate | 0.0037 | ** |
| Long Interval | Short Interval | 0.7997 |  |

# (I1) ANOVA - Retina TH Across Pre-Exposure Ages

| term | df | sumsq | meansq | statistic | p-value | signif. |
| --- | --- | --- | --- | --- | --- | --- |
| Ethanol | 1 | 0.1012 | 0.1012 | 2.0455 | 0.1558 |  |
| Caffeine | 2 | 0.2572 | 0.1286 | 2.5988 | 0.0794 |  |
| Pre.Exposure.Age | 3 | 0.8775 | 0.2925 | 5.9104 | 0.0009 | *** |
| Ethanol:Caffeine | 2 | 0.0394 | 0.0197 | 0.3976 | 0.6730 |  |
| Ethanol:Pre.Exposure.Age | 3 | 0.2633 | 0.0878 | 1.7736 | 0.1571 |  |
| Caffeine:Pre.Exposure.Age | 6 | 0.1743 | 0.0291 | 0.5871 | 0.7399 |  |
| Ethanol:Caffeine:Pre.Exposure.Age | 4 | 0.0711 | 0.0178 | 0.3590 | 0.8372 |  |
| Residuals | 100 | 4.9487 | 0.0495 | NA | NA | NA |

# (I2) Post hoc test for Pre-Exposure Age - Retina TH Across Pre-Exposure Ages

| group1 | group2 | adjusted p-value | signif. |
| --- | --- | --- | --- |
| 60 - 69 | 50 - 59 | 0.1509 |  |
| 70 - 99 | 50 - 59 | 0.6883 |  |
| 70 - 99 | 60 - 69 | 0.2336 |  |
| 140 - 149 | 50 - 59 | 0.0036 | ** |
| 140 - 149 | 60 - 69 | 0.0971 |  |
| 140 - 149 | 70 - 99 | 0.0078 | ** |

# (J1) ANOVA - Retina TH Across Time of Sacrifice

| term | df | sumsq | meansq | statistic | p-value | signif. |
| --- | --- | --- | --- | --- | --- | --- |
| Ethanol | 1 | 0.0593 | 0.0593 | 1.2757 | 0.2612 |  |
| Caffeine | 2 | 0.1484 | 0.0742 | 1.5975 | 0.2071 |  |
| Time.of.Sacrifice | 2 | 1.1704 | 0.5852 | 12.5973 | 0.0000 | *** |
| Ethanol:Caffeine | 2 | 0.0748 | 0.0374 | 0.8046 | 0.4499 |  |
| Ethanol:Time.of.Sacrifice | 2 | 0.0180 | 0.0090 | 0.1934 | 0.8245 |  |
| Caffeine:Time.of.Sacrifice | 4 | 0.3821 | 0.0955 | 2.0563 | 0.0915 |  |
| Ethanol:Caffeine:Time.of.Sacrifice | 4 | 0.2721 | 0.0680 | 1.4645 | 0.2180 |  |
| Residuals | 109 | 5.0634 | 0.0465 | NA | NA | NA |

# (J2) Post hoc test for Time of Sacrifice - Retina TH Across Time of Sacrifice

| group1 | group2 | adjusted p-value | signif. |
| --- | --- | --- | --- |
| Short Interval | Immediate | 0.0032 | ** |
| Long Interval | Immediate | 0.0000 | *** |
| Long Interval | Short Interval | 0.0528 |  |

# (L1) ANOVA - Brain GAD Across Pre-Exposure Ages

| term | df | sumsq | meansq | statistic | p-value | signif. |
| --- | --- | --- | --- | --- | --- | --- |
| Ethanol | 1 | 0.0208 | 0.0208 | 0.5395 | 0.4660 |  |
| Caffeine | 2 | 0.1216 | 0.0608 | 1.5791 | 0.2161 |  |
| Pre.Exposure.Age | 3 | 0.4805 | 0.1602 | 4.1596 | 0.0104 | * |
| Ethanol:Caffeine | 2 | 0.1338 | 0.0669 | 1.7368 | 0.1863 |  |
| Ethanol:Pre.Exposure.Age | 3 | 0.1290 | 0.0430 | 1.1169 | 0.3509 |  |
| Caffeine:Pre.Exposure.Age | 5 | 0.9248 | 0.1850 | 4.8033 | 0.0011 | ** |
| Ethanol:Caffeine:Pre.Exposure.Age | 4 | 0.2463 | 0.0616 | 1.5991 | 0.1888 |  |
| Residuals | 51 | 1.9640 | 0.0385 | NA | NA | NA |

# (L2) Post hoc test for Age - Brain GAD Across Pre-Exposure Ages

| group1 | group2 | adjusted p-value | signif. |
| --- | --- | --- | --- |
| 60 - 69 | 50 - 59 | 0.7760 |  |
| 70 - 99 | 50 - 59 | 0.1752 |  |
| 70 - 99 | 60 - 69 | 0.2933 |  |
| 140 - 149 | 50 - 59 | 0.0773 |  |
| 140 - 149 | 60 - 69 | 0.1752 |  |
| 140 - 149 | 70 - 99 | 0.7760 |  |

# (M) ANOVA - Brain GAD Across Time of Sacrifice

| term | df | sumsq | meansq | statistic | p-value | signif. |
| --- | --- | --- | --- | --- | --- | --- |
| Ethanol | 1 | 0.0034 | 0.0034 | 0.0673 | 0.7963 |  |
| Caffeine | 2 | 0.0259 | 0.0129 | 0.2578 | 0.7737 |  |
| Time.of.Sacrifice | 2 | 0.2698 | 0.1349 | 2.6903 | 0.0766 |  |
| Ethanol:Caffeine | 2 | 0.1755 | 0.0877 | 1.7499 | 0.1832 |  |
| Ethanol:Time.of.Sacrifice | 2 | 0.0139 | 0.0070 | 0.1386 | 0.8709 |  |
| Caffeine:Time.of.Sacrifice | 4 | 0.1319 | 0.0330 | 0.6578 | 0.6239 |  |
| Ethanol:Caffeine:Time.of.Sacrifice | 2 | 0.0322 | 0.0161 | 0.3212 | 0.7266 |  |
| Residuals | 56 | 2.8080 | 0.0501 | NA | NA | NA |

# (N1) ANOVA - Retina GAD Across Pre-Exposure Ages

| term | df | sumsq | meansq | statistic | p-value | signif. |
| --- | --- | --- | --- | --- | --- | --- |
| Ethanol | 1 | 0.1058 | 0.1058 | 0.3190 | 0.5734 |  |
| Caffeine | 2 | 0.6023 | 0.3011 | 0.9085 | 0.4064 |  |
| Pre.Exposure.Age | 3 | 4.4197 | 1.4732 | 4.4443 | 0.0056 | ** |
| Ethanol:Caffeine | 2 | 0.7848 | 0.3924 | 1.1838 | 0.3103 |  |
| Ethanol:Pre.Exposure.Age | 3 | 1.1065 | 0.3688 | 1.1127 | 0.3476 |  |
| Caffeine:Pre.Exposure.Age | 6 | 4.7570 | 0.7928 | 2.3917 | 0.0334 | * |
| Ethanol:Caffeine:Pre.Exposure.Age | 4 | 2.0864 | 0.5216 | 1.5735 | 0.1871 |  |
| Residuals | 101 | 33.4806 | 0.3315 | NA | NA | NA |

# (N2) Post hoc test for Pre-Exposure Age - Retina GAD Across Pre-Exposure Ages

| group1 | group2 | adjusted p-value | signif. |
| --- | --- | --- | --- |
| 60 - 69 | 50 - 59 | 0.0603 |  |
| 70 - 99 | 50 - 59 | 0.0165 | * |
| 70 - 99 | 60 - 69 | 0.7552 |  |
| 140 - 149 | 50 - 59 | 0.0221 | * |
| 140 - 149 | 60 - 69 | 0.7552 |  |
| 140 - 149 | 70 - 99 | 0.9721 |  |

# (O) Post Aligned Rank Transform ANOVA - Retina GAD Across Time of Sacrifice

| Term | df | Df.res | sumsq | Sum.Sq.res | statistic | p-value | signif. |
| --- | --- | --- | --- | --- | --- | --- | --- |
| Ethanol | 1 | 107 | 170.9068 | 162251.6 | 0.1127 | 0.7377 |  |
| Caffeine | 2 | 107 | 712.8563 | 161296.5 | 0.2364 | 0.7898 |  |
| Time.of.Sacrifice | 2 | 107 | 5997.4613 | 155538.8 | 2.0629 | 0.1321 |  |
| Ethanol:Caffeine | 2 | 107 | 3499.2396 | 157609.7 | 1.1878 | 0.3089 |  |
| Ethanol:Time.of.Sacrifice | 2 | 107 | 1549.3482 | 160332.6 | 0.5170 | 0.5978 |  |
| Caffeine:Time.of.Sacrifice | 4 | 107 | 9222.3232 | 150119.1 | 1.6433 | 0.1687 |  |
| Ethanol:Caffeine:Time.of.Sacrifice | 4 | 107 | 5979.9468 | 153993.3 | 1.0388 | 0.3908 |  |
